# Electrochemical and structural characterization of recombinant respiratory proteins of the acidophilic iron oxidizer *Ferrovum* sp. PN-J47-F6 suggests adaptations to the acidic pH at protein level

**DOI:** 10.3389/fmicb.2024.1357152

**Published:** 2024-02-07

**Authors:** Sophie R. Ullrich, Helena Fuchs, Charlotte Ashworth-Güth

**Affiliations:** ^1^Environmental Microbiology Group, Institute for Biological Sciences, TU Bergakademie Freiberg, Freiberg, Germany; ^2^Biohydrometallurgy Group, Institute for Biological Sciences, TU Bergakademie Freiberg, Freiberg, Germany; ^3^Salt and Mineral Chemistry Group, Institute for Inorganic Chemistry, TU Bergakademie Freiberg, Freiberg, Germany

**Keywords:** acidophiles, *Ferrovum*, HiPIP, c4 cytochrome, redox potential, cyclic voltammetry, Spectroelectrochemistry

## Abstract

The tendency of the periplasmic redox proteins in acidophiles to have more positive redox potentials (*E_m_*) than their homologous counterparts in neutrophiles suggests an adaptation to acidic pH at protein level, since thermodynamics of electron transfer processes are also affected by acidic pH. Since this conclusion is mainly based on the electrochemical characterization of redox proteins from extreme acidophiles of the genus *Acidithiobacillus*, we aimed to characterize three recombinant redox proteins of the more moderate acidophile *Ferrovum* sp. PN-J47-F6. We applied protein film voltammetry and linear sweep voltammetry coupled to UV/Vis spectroscopy to characterize the redox behavior of HiPIP-41, CytC-18, and CytC-78, respectively. The *E_m_*-values of HiPIP-41 (571 ± 16 mV), CytC-18 (276 ± 8 mV, 416 ± 2 mV), and CytC-78 (308 ± 7 mV, 399 ± 7 mV) were indeed more positive than those of homologous redox proteins in neutrophiles. Moreover, our findings suggest that the adaptation of redox proteins with respect to their *E_m_* occurs more gradually in response to the pH, since there are also differences between moderate and more extreme acidophiles. In order to address structure function correlations in these redox proteins with respect to structural features affecting the *E_m_*, we conducted a comparative structural analysis of the *Ferrovum*-derived redox proteins and homologs of *Acidithiobacillus* spp. and neutrophilic proteobacteria. Hydrophobic contacts in the redox cofactor binding pockets resulting in a low solvent accessibility appear to be the major factor contributing to the more positive *E_m_-values* in acidophile-derived redox proteins. While additional cysteines in HiPIPs of acidophiles might increase the effective shielding of the [4Fe-4S]-cofactor, the tight shielding of the heme centers in acidophile-derived cytochromes is achieved by a drastic increase in hydrophobic contacts (*A.f*. Cyc_41_), and by a larger fraction of aromatic residues in the binding pockets (CytC-18, CytC-78).

## 1 Introduction

Acid tolerance mechanisms in acidophilic microorganisms have been investigated to some extent with respect to the whole cell (Baker-Austin and Dopson, 2007; Slonczewski et al., 2009). However, less is known about the adaptational mechanisms at the protein level with respect to stability or function (Schäfer et al., 2004; Chi et al., 2007; Duarte et al., 2009; Cárdenas et al., 2010). Redox proteins of respiratory electron transfer chains present interesting candidates to study such adaptational mechanisms for two reasons. Firstly, they are exposed to external acidic pH and thus they likely need structural adaptations to maintain their native conformation (Baker-Austin and Dopson, 2007; Duarte et al., 2009). Secondly, *E_m_-values* of electron donor and acceptor couples are affected by pH. Consequently, the tuning of the redox proteins’ *E_m_*-values was postulated to present a necessary adaptation in order for them to effectively transfer electrons between these donor and acceptor couples (Bird et al., 2011).

In acidithiobacilli representatives, the *E_m_*-values of redox proteins of the electron transfer chain from ferrous iron to oxygen are found to be more positive than those of homologous proteins derived from neutrophiles (Giudici-Orticoni et al., 1999; Bird et al., 2011). For example, the *E_m_* of the HiPIPs Hip of *Acidithiobacillus ferridurans* (Bruscella et al., 2005) and Iro of *Acidithiobacillus ferrooxidans* (Yamanaka and Fukumori, 1995) are at least 100 mV more positive than those of HiPIPs in neutrophilic phototrophs. Similar observations have been reported for the blue copper protein rusticyanin (Ingledew and Cobley, 1980), the periplasmic c_4_ cytochromes Cyc1/Cyc_41_ (Cavazza et al., 1996) and CycA1 (Giudici-Orticoni et al., 2000), and the outer membrane cytochrome Cyc2 (Castelle et al., 2008). Their more positive *E_m_*-values appear to be attributed to (i) the more positive *E_m_*-values of the oxygen/water electron acceptor couple at acidic pH (1.12 Vat pH 2 vs. 0.82 V at pH 7) and (ii) to the dependency of *E_m_*-value the on solubility and chelators of the ferrous/ferric iron couple at a certain pH (Ingledew and Cobley, 1980; Bird et al., 2011; Ilbert and Bonnefoy, 2013).

Electrochemical studies on several redox proteins have revealed that their *E_m_* also depends on the pH during the measurement due to charge variations of specific surface exposed residues (Bian et al., 1996; Stephens et al., 1996; Capozzi et al., 1998). However, although this pH dependency has also been described for acidophile-derived redox proteins, their *E_m_*-values are generally more positive. For example, at pH 7 the *E_m_* of Hip (*A. ferridurans*) is 510 mV (Bruscella et al., 2005) while the *E_m_* of HiPIPs derived from the neutrophiles *Rhodoferrax fermentans* and *Rhodocyclus tenuis* are 351 mV (Hochkoeppler et al., 1995) and 330 mV (Meyer et al., 1983) respectively. Thus, it seems tempting to expect that specific structural features might have evolved in redox proteins in acidophiles which modulate their *E_m_* toward more positive values. Among the most prominent structural properties affecting the *E_m_* is the binding mode of the redox center within the binding pocket. This concerns both hydrophobic contacts (Carter et al., 1972; Dey et al., 2007), because they influence the solvent accessibility of the redox center (Parisini et al., 1999; de March et al., 2015), and electrostatic contacts such as hydrogen bonds between protein and redox center which affect the nucleophilic or electrophilic character of the redox center (Backes et al., 1991; Heering et al., 1995; Mao et al., 2003; Hosseinzadeh et al., 2016).

Being a more moderate acidophile, *Ferrovum* sp. PN-J47-F6 presents an interesting model to study acid adaptational mechanisms due to its exceptional position between neutrophiles and more extreme acidophiles (Ullrich et al., 2016a,b; Grettenberger et al., 2020). Our recent study on the recombinant redox protein candidates of *Ferrovum* sp. PN-J47-F6 suggested the high-potential iron–sulfur protein HiPIP-41 and the two c_4_ cytochromes CytC-18 and CytC-78 to be likely candidates of the electron transfer chain from ferrous iron to oxygen (Ullrich et al., 2023). Building on this earlier study, our present study focused, firstly, on collecting evidence on whether the redox properties of HiPIP-41, CytC-18 and CytC-78 might reflect the unique position of their host *Ferrovum* sp. PN-J47-F6 between neutrophiles and acidophiles. Secondly, we were interested in whether their redox properties are attributed to certain structural features. The prerequisite of studying such correlations between structure and function is the parallel availability of electrochemical data and structural information. Therefore, we combined an experimental and a computational approach. We determined the *E_m_*-values of HiPIP-41, CytC-18 and CytC-78 of *Ferrovum* sp. PN-J47-F6 using potentiometric approaches and compared them to *E_m_*-values of homologous proteins derived from neutrophiles and acidophiles with the aim to evaluate the trend of more positive *E_m_*-values in acidophile-derived redox proteins. Our secondary aim was to identify structural features with respect to the redox cofactor binding pocket and solvent accessibility that might contribute to the more positive *E_m_*-values. Therefore, we analyzed and compared experimentally solved structures of homologous redox proteins and structural models of the *Ferrovum*-derived recombinant redox proteins predicted by the D-i-Tasser suite.

## 2 Materials and methods

### 2.1 Heterologous production and purification of recombinant redox proteins

Recombinant His-tagged HiPIP-41, CytC-18, and CytC-78 derived from *Ferrovum* sp. PN-J47-F6 were produced, purified and concentrated as described previously (Ullrich et al., 2023).

### 2.2 Determination of the redox potential

If not stated otherwise all experiments were conducted at 25°C in sodium citrate phosphate buffer (41.4 mM Na_2_HPO_4_, 79.3 mM citric acid, pH 3.1). The experimental set-up is shown in Supplementary Figure 1.

#### 2.2.1 Protein film voltammetry

The redox potential of HiPIP-41 was determined directly via classic cyclic voltammetry (CV). Control potential scans of the buffer without HiPIP-41 showed redox peaks. HiPIP-41 (600 μM) was applied directly as thin protein film on the freshly polished surface (0.07 cm^2^) of a glassy carbon working electrode. The working electrode was inserted into a Slide-A-Lyzer Mini Dialysis Unit (MWCO 3.5 kDa; Pierce) in order to prevent protein dilution during CV measurements. The dialysis unit was inserted into a buffer filled glass vessel together with the platinum counter electrode and the Ag/AgCl (in 3 M NaCl) reference electrode. CV experiments were conducted using a Gamry Interface1000 potentiostat and the Gamry Framework software. Data was collected between potential limits of 200 and 900 mV vs. SHE with potential scan rates of 10, 50 and 100 mV/s which were applied in direct succession to the assay. The *E_m_* of HiPIP-41 was determined based on the potentials of its oxidative and reductive peaks. A correction factor of 197 mV was used to convert redox potentials from vs. Ag/AgCl (3 M NaCl) to vs. standard hydrogen electrode (SHE).

#### 2.2.2 Linear sweep voltammetry coupled to UV/Vis spectroscopy

Concentrates of recombinant cytochromes CytC-18 and CytC-78, respectively, were diluted in buffer solution within a spectroelectrochemical quartz glass cuvette (PINE Research). In order to facilitate electron transfer between the cytochrome and the working electrode surface the redox mediators potassium ferricyanide and phenanzine methosulfate (PMS) were added in 2-fold excess. Our choice of redox mediators was based on fact that their absorption spectra did not overlap with the α-, β- and γ-peaks of the cytochromes and because their *E_m_*-values are well within the indented potential limits. Moreover, PMS has already been demonstrated as suitable redox mediator in redox titrations of c-type cytochromes (Carpenter et al., 2020), while ferricyanide has been shown to oxidize CytC-18 and CytC-78 in a biochemical redox assay (Ullrich et al., 2023). Stocks of buffer and redox mediators were treated with N_2_ to reduce remaining O_2_ content prior use. The final electrochemical assay of 450 μL contained 20 μM of CytC-78 and approx. Forty micrometer of each redox mediator or 36 μM CytC-18 and 72 μM of each redox mediator, respectively. A platinum honeycomb working electrode (PINE Research) and an Ag/AgCl reference electrode (in 3 M KCl) were inserted into the cuvette and connected to a Gamry Interface1000 potentiostat. The cuvette was placed in a Jasco V-670 UV/Vis spectrometer. The open circuit potential of the reaction mix was determined to set the initial potential for the reductive sweep. Before each potential sweep the initial potential was held for 120 s. Bernhardt, 2023 demonstrated the suitability of potential sweep rates between 0.1 and 0.6 mV/s for horse heart cytochrome c. Based on results on horse heart cytochrome c (Bernhardt, 2023) using a similar experimental set-up, we ran reductive and oxidative potential sweeps in sequence between 450 mV and − 50 mV vs. Ag/AgCl at 0.1, 0.15, and 0.2 mV/s with a step size of 0.5 mV for each assay. UV/Vis spectra between 390 and 590 nm were recorded every 10 mV during the potential sweeps. Potential absorption curves were determined for the α-, β-, and γ-peaks of the respective cytochrome within Prism Graph Pad 6. Boltzmann sigmoidal fit was applied to determine the infliction points. *E_m_*-values were calculated as the average of the infliction points of corresponding oxidative and reductive potential sweeps. The redox potential vs. standard hydrogen electrode (SHE) was calculated by adding 200 mV to the potential against the Ag/AgCl (3 M KCl) reference electrode.

### 2.3 Prediction and analysis of structural models

Structural models of the mature wildtype protein sequences of HiPIP-41 (WP_067495359), CytC-18 (WP_067493319), and CytC-78 (WP_229347545) were predicted using the D-i-Tasser suite (Deep-learning based Iterative Threading ASSEmbly Refinement; Yang and Zhang, 2015; Zheng et al., 2022, 2023). N-terminal signal peptides were predicted using SignalP5.0 (Almagro Armenteros et al., 2019) and omitted from the protein sequence. Structural models of the HiPIP of *Rhodocyclus tenuis* (1Isu), Cyc_41_ of *Acidithiobacillus ferrooxidans* (1h1o) and c_4_ of *Pseudomonas strutzeri* (1 m70) were retrieved from the Protein Database (PDB). The structural model of Hip of *Acidithiobacillus ferridurans* (UniProt ID: Q93MF8) was retrieved from the AlphaFold protein Structure Database (Jumper et al., 2021). Structural models were visualized using Chimera 1.15 (Pettersen et al., 2004). Superimposition of the HiPIP structural models was achieved using MatchMaker implemented in Chimera 1.15 (Meng et al., 2006). Hydrophobic contacts and hydrogen bonds at the domain:domain interfaces of c_4_ cytochromes and within the cofactor binding pockets were predicted by LigPlot^+^ (Wallace et al., 1995; Laskowski and Swindells, 2011) and amended by manual inspection of the structural models.

## 3 Results

### 3.1 Electrochemical characterization of the *Ferrovum*-derived recombinant redox proteins

The electrochemical characterization of the recombinant *Ferrovum*-derived redox proteins HiPIP-41, CytC-18 and CytC-78 aimed to determine the *E_m_*-values of their cofactors. The different size of the native redox proteins and the different nature of their cofactors required different approaches for their electrochemical characterization.

#### 3.1.1 Determination of the redox potential of HiPIP-41 using protein film voltammetry

**Figure 1:**
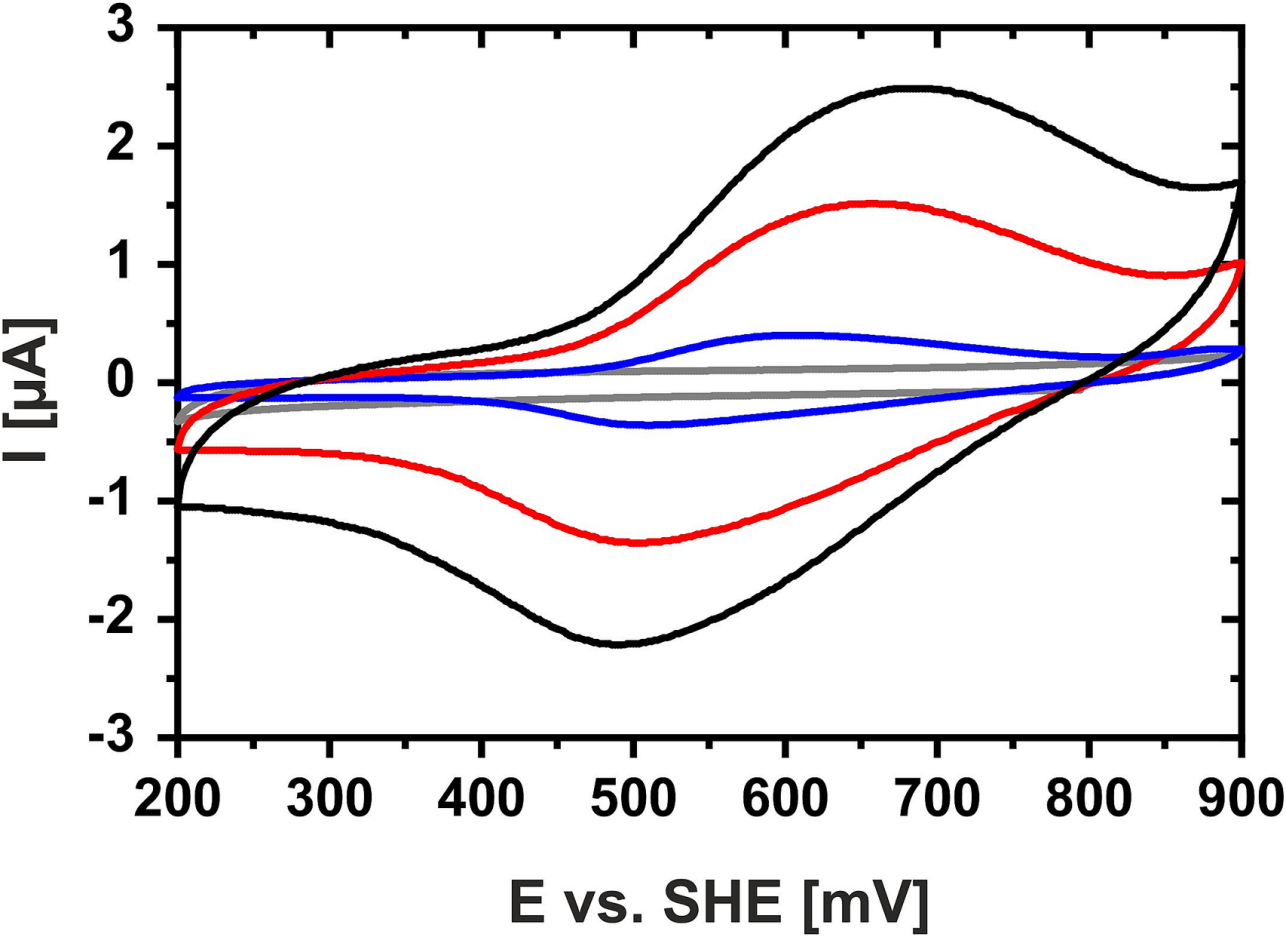
Cyclic voltammogram of HiPIP-41 in sodium phosphate citrate buffer (pH 3.1). Cyclic voltammograms of HiPIP-41 in sodium citrate phosphate buffer, pH 3.1 are shown for scan rates of 100 mV/s (black curve), 50 mV/s (red curve) and 10 mV/s (blue curve), respectively, and between potential limits of 200 and 900 mV vs. SHE. The voltammogram of the buffer control without HiPIP-41 is shown in gray. HiPIP-41 was applied as a thin protein film directly onto the surface of the glassy carbon working electrode at a final concentration of 600 μM. The redox assay was carried out in sodium citrate phosphate buffer using an Ag/AgCl as reference electrode and a platinum counter electrode.

Cyclic voltammetry of a concentrated HIPIP-41 film applied directly onto the working electrode surface proved suitable to determine the *E_m_* of HiPIP-41. The cyclic voltammograms of HiPIP-41 taken at three different scan rates (10, 50 and 100 mV/s) show the fully reversible character of the electrochemical reduction and oxidation of HiPIP-41 (Figure 1). The averaged *E_m_* was calculated to be 571 mV ± 16 mV vs. SHE based on the curves of all three potential scan rates. This is in accord with the very positive *E_m_*-values of HiPIPs found in acidophilic chemolithoautotrophs (Table 1). Hip of *A. ferridurans* is involved in periplasmic electron transfer during oxidation of reduced sulfur compounds (Bruscella et al., 2005; Quatrini et al., 2006) and has an *E_m_* of 550 mV (Bruscella et al., 2005). The HiPIP Iro of *A. ferrooxidans* has an *E_m_* of 633 mV (Yamanaka and Fukumori, 1995) and has been suggested to be involved in ferrous iron oxidation (Fukumori et al., 1988). Electrochemically characterized HiPIPs of neutrophilic bacteria are restricted to the phototrophic bacteria *Rhodoferrax fermentans*, *Rhodocyclus tenuis* 2,761, *Rhodopseudomonas palustris* TIE-1 and *Halorhodospira halophila*. Their *E_m_*-values lie in a range of 50 mV (Iso-HiPIP II, *H. halophila*) and 450 mV (PioC, *R. palustris*) but are generally more negative than those of the acidophiles (Table 1). Among the phototrophs, PioC of *R. palustris* has the most positive *E_m_* (450 mV). PioC serves as periplasmic electron shuttle between an iron oxidizing outer membrane protein complex PioAB and the reaction center of the photosystem II (Bird et al., 2014). The HiPIPs of the other neutrophilic representatives mediate the periplasmic electron transfer between an inner membrane protein complex and the reaction center of photosystem II (Hochkoeppler et al., 1995). Apparently, the *E_m_* of HiPIPs is attributed to both, the nature of the electron transfer process and consequently its interaction partners as well as the prevailing pH of the periplasm.

**Table 1:** Overview of redox potentials of HiPIP-41 and homologous proteins in other bacteria.

Taxonomic affiliation	Original host	Protein name	*E_m_*, _pH_ vs. SHE	Methodological approach	References
*β-Proteobacteria*	*Ferrovum* sp. PN-J47-F6	HiPIP-41	*E_m_*, _3.1_ = 571 mV (± 16 mV)	Cyclic voltammetry (protein film)	This study
	*Rhodoferrax fermentans*	HiPIP	*E_m_*, _7_ = 351 mV	Optical redox titration	Hochkoeppler et al. (1995)
	*Rhodocyclus tenuis* 2761	HiPIP	*E_m_*, _7_ = 330 mV	Biochemical assay	Meyer et al. (1983)
*α-Proteobacteria*	*Rhodopseudomonas palustris* TIE-1	PioC	*E_m_*, _9_ = 450 mV	Cyclic voltammetry	Bird et al. (2014)
*γ-Proteobacteria*	*Halorhodospira halophila*	Iso-HiPIP II	*E_m_*, _7_ = 50 mV	Optical redox titrations	Lieutaud et al. (2005)
*Acidithiobacillia*	*Acidithiobacillus ferridurans* ATCC 33020	Hip	*E_m_*, _7_ = 510 mV (± 5 mV)	Cyclic voltammetry, square wave voltammetry (protein film)	Bruscella et al. (2005)
		Hip	*E_m_*, _2_ = 550 mV (± 10 mV)	Cyclic voltammetry, square wave voltammetry (protein film)	Bruscella et al. (2005)
	*Acidithiobacillus ferrooxidans*	Iro	*E_m_*, _3.5_ = 633 mV		Yamanaka and Fukumori (1995)

The methodological approach for redox potential determination is given along with the pH of the electrochemical assay (*E_m_*, _pH_), the reference to the study and a general taxonomic affiliation of the HiPIP’s original host. HiPIP-41 of *Ferrovum* sp. PN-J47-F6 and Iro of *Acidithiobacillus ferrooxidans* are proposed to be involved in ferrous iron oxidation while Hip of *Acidithiobacillus ferridurans* is proposed to be involved in reduced sulfur compounds oxidation. The HiPIPs in the other bacteria are involved in electron transfer to photosystem II. *Ferrovum* sp. PN-J47-F6 and *Acidithiobacillus* spp. present acidophilic bacteria while the phototrophic bacteria are neutrophiles.

#### 3.1.2 Determination of the redox behavior of CytC-18 and CytC-78 using a spectroelectrochemical approach

Our previous biochemical redox assays demonstrated the reversibility of oxidation and reduction of CytC-18 and CytC-78 (Ullrich et al., 2023). However, cyclic voltammetry of a cytochrome protein film was not feasible for CytC-18 and CytC-78, because of the irreversibility of the electrochemical oxidation or reduction, respectively (data not shown). With respect to observations reported for the green copper protein AcoP of *A. ferrooxidans*, it is possible that CytC-18 and Cytc-78 also underwent irreversible conformational changes during contact with the electrode surface which impaired their redox activity (Wang et al., 2018). Therefore, we chose a mediated spectroelectrochemical approach to further characterize the redox behavior of the two cytochromes. This approach combined linear sweep voltammetry and the simultaneous recording of UV/VIS spectra. Thereby, the applied potential was changed at very slow rates in presence of redox mediators facilitating the electron transfer between the cytochromes and the working electrode while the cytochrome’s redox state was assessed by determination of the absorption intensity of the α-, β-, γ-peaks.

**Figure 2:**
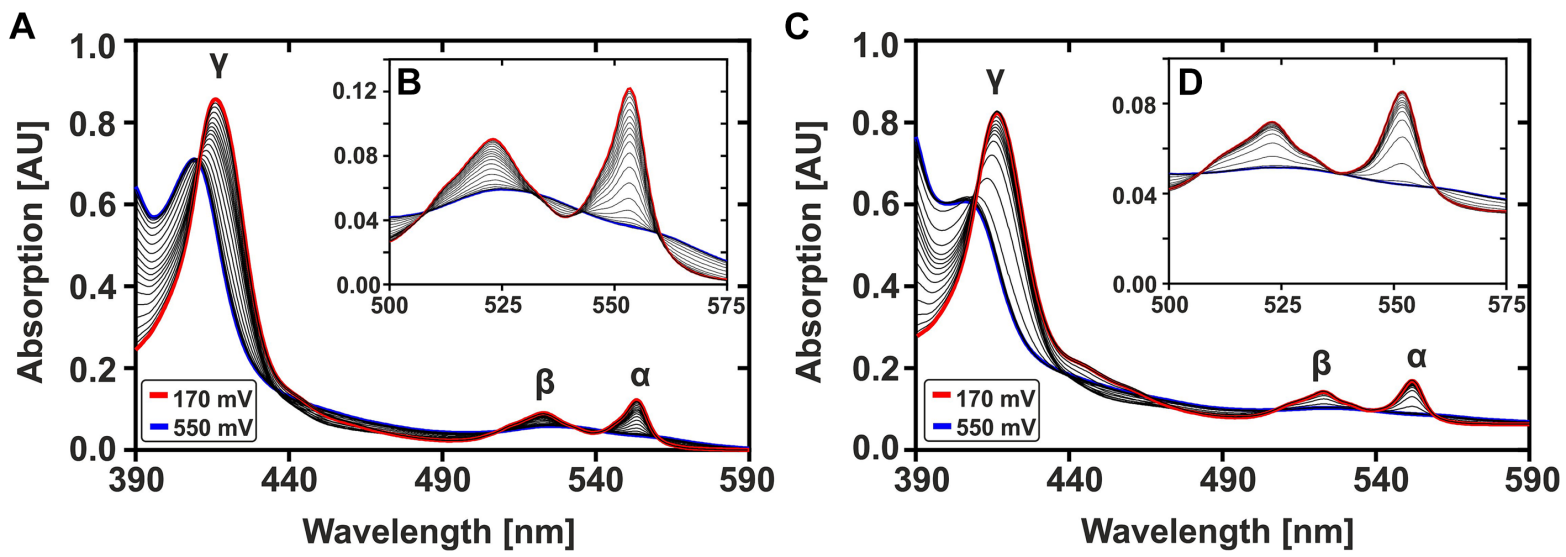
Potential-dependent absorption spectra of CytC-18 **(A,B)** and CytC-78 **(C,D)**. Absorption spectra between 390 and 590 nm were recorded during the oxidative potential sweep from 170 to 550 mV vs. SHE at 0.1 mV/s. For each cytochrome, a total of 20 spectra was stacked to visualize the redox transition between fully reduced state at 170 mV (red curve) and fully oxidized state at 550 mV (blue curve). The typical cytochrome c absorption maxima at 552 nm (α-peak), 525 nm (β-peak), and at ~420 nm (γ-peak) are indicated. The inlet enlarges the α- and β-peaks **(B,D)**. The redox assay was carried out in a specialized quartz glass cuvette using a platinum working electrode in honeycomb design, an Ag/AgCl (3 M KCl) reference electrode and a platinum counter electrode. The assay contained 36 μM CytC-18 or 20 μM CytC-78, respectively, in the presence of two-fold excess of sodium ferricyanide and phenazine methosulfate serving as redox mediators in N_2_-treated sodium citrate phosphate buffer (pH 3.1).

Figure 2 shows the complete oxidation of CytC-18 (A, B) and CytC-78 (C, D) during a potential sweep from 170 mV to 550 mV vs. SHE at 0.1 mV/s. During this oxidative potential sweep the intensity of the α- and β-peaks decrease while the γ-peaks shift to shorter wavelengths and decreases in intensity. The stacking of the absorption spectra creates a unique pattern for each of the two cytochromes, because the rate of absorbance intensity change per 20 mV varied over the potential sweep. There are potential windows when the absorbance intensity is only slightly changing between sequential spectra as well as potential windows where the intensity is changing to a larger extent. For CytC-18 (Figures 2A,B) the potential windows with faster and slower absorbance changes are clearly alternating which is likely to be attributed to the two heme centers that are oxidized sequentially rather than simultaneously. This behavior was also observed for CytC-78 (Figures 2C,D), but it appears to occur less uniformly between the potential limits. Instead, there is a potential window between 390 and 470 mV vs. SHE in which CytC-78 oxidizes to a larger extent indicated by the higher absorption changes between subsequent 20 mV potential steps.

**Figure 3:**
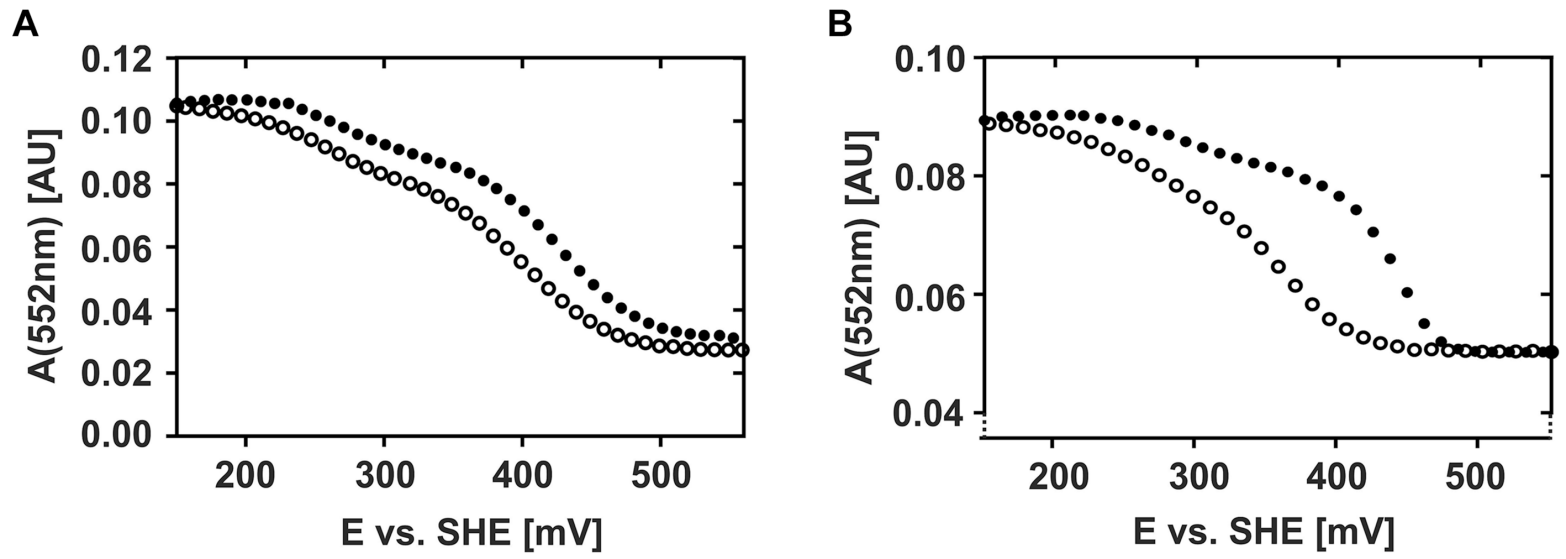
Potential-absorption plots of CytC-18 **(A)** and CytC-78 **(B)**. The intensity of the respective α-peak is plotted against the applied potential vs. SHE. The cytochromes were electrochemically oxidized (closed circles) or reduced (open circles) at a platinum honeycomb working electrode in the presence of two-fold excess of the redox mediators phenanzine methosulfate and ferricyanide. The concentration of the cytochromes was 36 μM of CytC-18 **(A)** or 20 μM of CytC-78 **(B)**, respectively. The potential sweep rate was 0.15 mV/s for each scan. Absorption was measured every 10 mV **(A)** or 12 mV **(B)**, respectively.

These observations are even more clearly reflected when plotting the absorption intensities of the respective α-, β-, and γ-peaks against the applied potential (Supplementary Figure 2) suggesting that CytC-18 and CytC-78 are characterized by a unique redox behavior. Representatively, Figure 3 depicts the individual redox behavior of CytC-18 (A) and CytC-78 (B) as functions of the absorption intensities of their respective α-peaks in dependence of the applied potential at a sweep rate of 0.15 mV/s. The resulting curves for the two cytochromes are characterized by a double sigmoidal shape reflecting two apparent redox transitions for the two hemes in each of the cytochromes. The lower plateau of absorption intensity corresponds with higher potentials and the fully oxidized state of the cytochromes, while the upper absorption intensity plateau corresponds with lower potentials and the fully reduced state of the cytochromes. An intermediary plateau is only slightly indicated by the slower absorption intensity changes. This intermediate plateau is more clearly visible for CytC-18 independent from the direction of the potential sweep (Figure 3A). In the case of CytC-78 this intermediary plateau is more clearly shaped in the curves of the oxidative potentials sweeps (Figure 3B). For both cytochromes the lower potential transition corresponds to only 25% of the total intensity difference between the fully reduced and fully oxidized state while the higher potential transition corresponds to 75%. This leads to the different steepness of the two sigmoidal curve areas in the lower or higher potential window, respectively.

Apart from the overall similar shape of the curves for both cytochromes, there are two features that again suggest an individual redox behavior of two *Ferrovum*-derived cytochromes: (i) the different extents of hysteresis between the curves of the oxidative and reductive sweeps and (ii) the size of the potential window required for the full redox transition. With respect to hysteresis, the curves of oxidative and reductive potential sweeps are very similar in shape and course for CytC-18 (Figure 3A). In the case of CytC-78 hysteresis increases with the potential sweep rate from 0.1 to 0.2 mV/s (Supplementary Figure 2B). Furthermore, the potential window for full redox transition is generally smaller for CytC-78 than for CytC-18 with approx. 280 mV vs. approx. 330 mV, respectively, (Supplementary Table 1A). This observation might be attributed to the smaller size of the potential window corresponding with the higher potential transition in CytC-78 compared to CytC-18 which also results in even steeper sigmoidal curves for the higher potential transition in CytC-78.

We calculated the *E_m_*-values for the two heme centers of CytC-18 and CytC-78 based on the inflection points of the lower potential transition (lower potential heme) and the higher potential transition (higher potential heme) for the different potential sweep rates and for the curves of α-, β- and γ-peaks (Supplementary Table 1B). Since the deviation of the calculated *E_m_*-values was low for the different sweep rates and absorption peaks, we calculated the mean values for the lower and higher potential hemes of CytC-18 and CytC-78 to compare them to the available *E_m_*-values of homologous c_4_ cytochromes of neutrophiles and acidophiles (Table 2). For CytC-18 we determined *E_m_*-values of 276 mV (± 8 mV) for the lower potential heme and 416 mV (± 2 mV) for the higher potential heme and 308 mV (± 7 mV) and 399 mV (± 7 mV) for CytC-78, respectively. The Δ*E_m_* of the two heme centers is smaller in CytC-78 than in CytC-18 (91 vs. 140 mV), which is in accordance with the smaller potential window to achieve the full redox transition in CytC-78. In comparison to c_4_ cytochromes derived from neutrophiles (*Pseudomonas* spp., *Pseudoalteromonas haloplanktis*, *Azotobacter vinelandii*, *Vibrio cholerae*) the *E_m_*-values of both *Ferrovum*-derived cytochromes are at least 50 mV more positive (Table 2). On the other hand, they are approx. 70 mV more negative than the *E_m_*-values of c_4_ cytochromes derived from the more extreme acidophiles *Acidithiobacillus* spp. The Δ*E_m_* of the lower and higher potential heme ranges from 54 to 110 mV, highlighting the unexpectedly large Δ*E_m_* of the CytC-18 heme centers.

**Table 2:** Summary of determined redox potentials of the c_4_ cytochromes CytC-18, CytC-78, and homologous proteins.

Taxonomic affiliation	Original host	Protein name	*E_m_*, _pH_ vs. SHE	Methodological approach	References
*β-Proteobacteria*	*Ferrovum* sp. PN-J47-F6	CytC-18	*E_m_*, _3.1_ = 276 mV (± 8 mV) *E_m_*, _3.1_ = 416 mV (± 2 mV)	Spectroelectrochemical voltammetry	This study
		CytC-78	*E_m_*, _3.1_ = 308 mV (± 7 mV) *E_m_*, _3.1_ = 399 mV (± 7 mV)	Spectroelectrochemical voltammetry	This study
*γ-Proteobacteria*	*Pseudomonas aeruginosa*	*P.a.* Cyt c_4_	*E_m_*, _7.4_ = 322 mV (± 2 mV)	Spectroelectrochemical voltammetry	Carpenter et al. (2020)
	*P. aeruginosa*	Cyt. c_4_	*E_m_*, _7_ = 268 mV *E_m_*, _7_ = 322 mV	Optical redox titration	Leitch et al. (1985)
	*Pseudomonas strutzeri*	*P.s.* cyt c_4_	*E_m_*, _7.4_ = 258 mV (± 4 mV) *E_m_*, _7.4_ = 364 mV (± 6 mV)	Spectroelectrochemical voltammetry	Carpenter et al. (2020)
	*Pseudoalteromonas haloplanktis*	Cyt. C_4_	*E_m_*, _7.5_ = 322 mV	Cyclic voltammetry (protein in solution)	Di Rocco et al. (2008)
	*Azotobacter vinelandii*	Cyt. c_4_	*E_m_*, _7_ = 263 mV *E_m_*, _7_ = 317 mV	Optical redox titration	Leitch et al. (1985)
	*Vibrio cholerae*	Cyt. C_4_	*E_m_*, _7_ = 240 mV *E_m_*, _7_ = 340 mV	Cyclic voltammetry (protein film)	Chang et al. (2010)
*Acidithiobacillia*	*A. ferridurans* ATCC 33020	Cyc1	*E_m_*, _3_ = 385 mV (± 20 mV) *E_m_*, _3_ = 480 mV (± 20 mV)	Optical redox titration	Cavazza et al. (1996)
	*Acidithiobacillus ferrooxidans*	Cyc41	*E_m_*, _4.6_ = 350 mV (± 10 mV) *E_m_*, _4.6_ = 460 mV (± 10 mV)	Cyclic voltammetry, square wave voltammetry	Malarte et al. (2005)
	*A. ferriphilus*	Cyt. c_4_ (CycA1)	*E_m_*, _4.5_ = 430 mV (± 20 mV) *E_m_*, _4.5_ = 510 mV (± 20 mV)	Optical redox titration	Giudici-Orticoni et al. (2000)

For each cytochrome the determined redox potentials of the two heme centers are given along with the methodological approach of their determination, the pH of the electrochemical assay (*E_m_*, _pH_), the reference to the study and a general taxonomic affiliation of the cytochrome’s original host. *Ferrovum* sp. PN-J47-F6 and *Acidithiobacillus* spp. present acidophilic bacteria while the other bacteria are neutrophiles.

Since the *E_m_*-values of two different classes of redox proteins showed similar tendencies when compared to homologous proteins of acidophiles and neutrophiles, we aimed at identifying potential structural features that might contribute to this adaptation at protein level. Therefore, we analyzed the cofactor binding pockets in predicted structural models and compared them to available experimentally solved structures or structural models of homologous proteins with available *E_m_* data.

### 3.2 Analysis of redox center environments of HiPIP-41 and comparison to homologous HiPIPs

The [4Fe-4S]-cofactor in HiPIPs is covalently bound by four highly conserved cysteine residues and is stabilized in a hydrophobic binding pocket with aromatic residues playing a fundamental role in electron transfer and *E_m_* modulation (Agarwal et al., 1995; Iwagami et al., 1995; Bian et al., 1996; Parisini et al., 1999; Liu et al., 2014). Our previous analysis of the homology-based structure of HiPIP-41 has already suggested that the [4Fe-4S]-cofactor is surrounded by a high number of hydrophobic and aromatic residues (Ullrich et al., 2023). Thus, the new structural model of HiPIP-41 based on a combined approach of homology modeling and a deep learning algorithm was compared to the AlphaFold-predicted structure of Hip of the acidophile *A. ferrooxidans* and the experimentally solved structure of the HiPIP of *R. tenuis* (PDB: 1Isu). Hip and HiPIP 1Isu were chosen for this comparative approach because they have already been electrochemically characterized (Table 1). Our analysis focused on conserved and unique structural features with special regard to residue candidates involved in tuning the *E_m_*-values.

**Figure 4:**
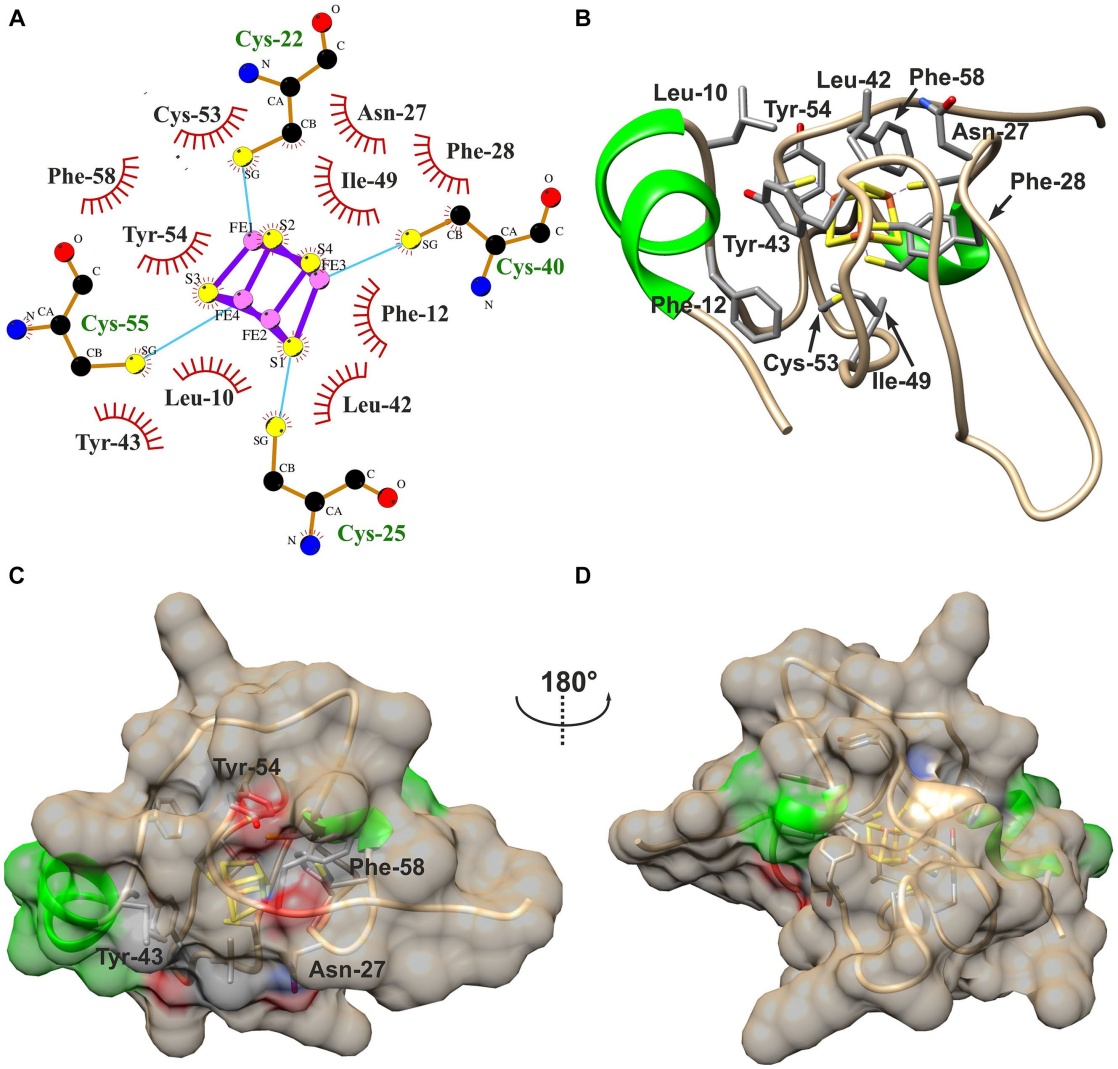
Hydrophobic contacts to the [4Fe-4S]-cofactor in HiPIP-41. **(A)** The hydrophobic contacts of the cofactor-binding pocket are visualized as ligplot predicted by LigPlot^+^ and amended after manual inspection of the structure model. Hydrophobic contacts are depicted by red eyelashes while other bonds are shown as blue solid lines between atoms. **(B)** Residues corresponding with the ligplot are shown in the structural model by three-letter code and position number. Residues are colored by element (carbon – gray, oxygen – red, nitrogen – blue, sulfur – yellow) **(C)** The [4Fe-4S]-cofactor is buried deep within the protein and shielded from the outside. The surface of HiPIP-41 is colored by secondary structural element (helix – green, coil – tan). The residues depicted in **(B)** have influence on the surface as is indicated by colored patches on the surface. The colors refer to the element coloring of side chains or peptide bonds of the respective residues. **(D)** The structure is shown from a different view as indicated by the screw axis.

Hydrophobic contacts between the protein and the cofactor in the three HiPIPs were predicted using LigPlot^+^ with subsequent evaluation by manual inspection of their structural models. The [4Fe-4S]-cofactor in HiPIP-41 is surrounded by 10 hydrophobic residues of which five are aromatic (Figures 4A,B). The high number of hydrophobic contacts completely shields the cofactor from the surrounding solvent (Figures 4C,D). However, some of these residues not only contribute to the hydrophobic character of the binding pocket but also to the surface properties of HiPIP-41. The hydroxyl group of Tyr-54, for example, is oriented toward the surface while the aromatic ring shields the cofactor (Figure 4C). The polar sidechain of the Asn-27 is similarly oriented toward the surface while the CH_2_-group is positioned toward the protein core (Figure 4D). In the case of Tyr-43 and Phe-58 the oxygen moieties of their peptide bonds are pointing toward the surface (Figure 4C).

The cofactor in HiPIP 1Isu is surrounded by a similar number of hydrophobic (10) and aromatic residues (5) (Supplementary Figures 3A,B) while the ten hydrophobic contacts to the Hip cofactor include only four aromatic residues (Supplementary Figures 3C,D). Similarly to HiPIP-41, the cofactors of Hip and HiPIP 1Isu are completely buried within the protein cores and are not accessible for the surrounding solvent (Supplementary Figures 3E,F). The superimposition of all three structural models shows the high structural similarity of all HiPIPs where HiPIP-41 and HiPIP 1Isu even share the short N-terminal α-helix (Supplementary Figure 4). The high proline content of Hip was reported to interfere with secondary structural elements (Nouailler et al., 2006), which might explain the slightly lower structural similarity to the other two HiPIPs. Despite the generally low sequence similarities of less than 40% between the three HiPIPs, many of the residues with hydrophobic contacts to the cofactor were identified at identical positions (Supplementary Figures 4A–C). A remarkable exception presents position 53 (1Isu numbering), where HiPIP-41 harbors an additional cysteine residue while HiPIP 1Isu and Hip have glycine residues. Two other positions are also noteworthy, because only one of the three HiPIPs possesses an aromatic residue while the other two harbor aliphatic residues at the same position: the first is Phe-10 in HiPIP 1Isu and the second is Tyr-43 in HiPIP-41. Otherwise four of five aromatic residues with hydrophobic contacts to the cofactor are highly conserved between all three structures (Supplementary Figure 4C).

However, the only clearly distinguishing feature of the two acidophile-derived HiPIPs Hip and HiPIP-41 present the two additional cysteine residues not involved in cofactor coordination (Supplementary Figure 4D). While Cys-52 and Cys-84 of Hip were found to form a structure stabilizing disulfide bond (Nouailler et al., 2006), the role of Cys-50 and Cys-53 of HiPIP-41 has not yet been elucidated (Ullrich et al., 2023). The orientation of Cys-53 toward the cofactor in HiPIP-41 together with its restricted presence in *Ferrovum* spp. HiPIPs (Ullrich et al., 2023) seems to be a rather striking. Still, it remains so far unclear whether these additional cysteines might contribute to the at least 240 mV more positive *E_m_* in the HiPIPs of the acidophiles.

### 3.3 Analysis of redox center environments of CytC-18 and CytC-78 and comparison to homologous c_4_ cytochromes

The electrochemical characterization of CytC-18 and CytC-78 showed (i) that the potential window for the full redox transition is smaller for CytC-78 which corresponds with the smaller Δ*E_m_* of its two heme centers and (ii) that the hysteresis between the oxidative and reductive potential sweeps is smaller in CytC-18. Hence, although the *E_m_*-values of the two *Ferrovum*-derived cytochromes are quite similar, both are characterized by an individual redox behavior. Earlier studies suggested that both, the heme-to-heme positioning and the heme:protein contacts affect the intramolecular electron transfer (Kadziola and Larsen, 1997; Abergel et al., 2003; de March et al., 2015). Aiming to collect evidence for correlations between structure and properties of CytC-18 and CytC-78, we focused our analyses on their hydrogen bond networks at the domain:domain interface, hydrophobic contacts in the heme-binding pockets and the solvent accessibility of their heme centers. Moreover, these analyses provided the basis for the subsequent comparison to available structures of the homologous c_4_ cytochromes of the neutrophile *P. strutzeri* (PDB: 1 m70; *P.s.* c_4_) and of Cyc_41_ of the acidophile *A. ferrooxidans* (PDB: 1h1o; *A.f.* Cyc_41_) which have also been electrochemically characterized.

#### 3.3.1 Contacts at the domain:domain interface and in the heme-binding pockets of CytC-18 and CytC-78

**Figure 5:**
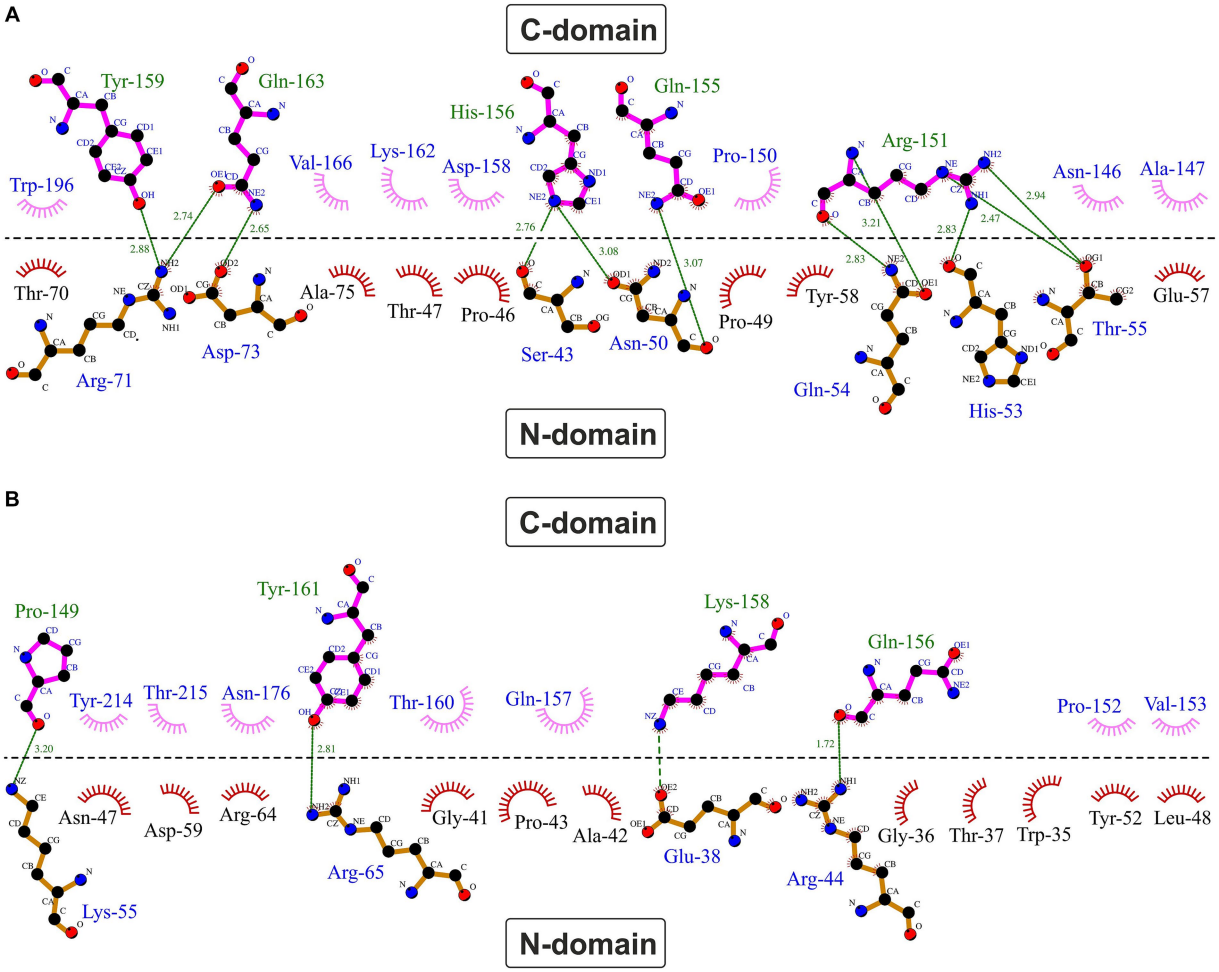
Hydrophobic contacts and hydrogen bonds at the domain:domain interface in CytC-18 **(A)** and CytC-78 **(B)**. The dimplots were calculated using LigPlot^+^. The horizontal line indicates the domain:domain interface between the N- and C-terminal domains. Hydrogen bonds are shown in green broken lines with distances between the bonding atoms in Å. Hydrophobic contacts are represented as red (N-terminal domains) or pink (C-terminal domains) eyelashes.

**Figure 6:**
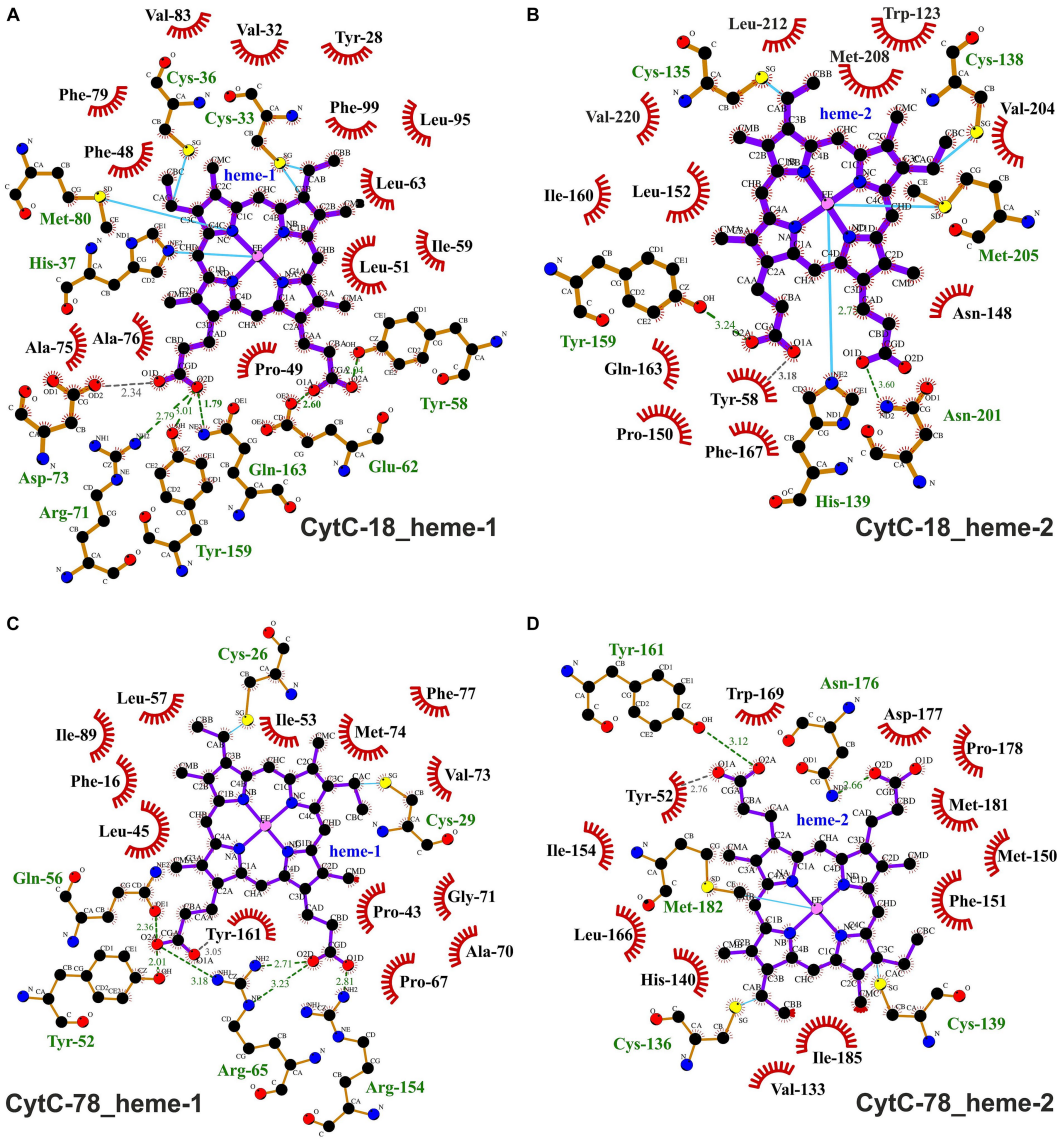
Ligplots visualizing hydrophobic contacts and hydrogen bonds between the heme ligands and the protein chains of CytC-18 **(A,B)** and CytC-78 **(C,D)**. Hydrogen bonds and hydrophobic contacts between the two heme c ligands and the protein chains of CytC-18 and CytC-78 were predicted using LigPlot^+^. The heme c ligands of the N-terminal domains are termed hemes-1 **(A,C)**, while those of the C-terminal domains are termed hemes-2 **(B,D)**. Hydrophobic contacts are depicted by red eyelashes while hydrogen bonds are shown as green broken lines with the distances between the bonding atoms given in Å. Other bonds are shown as blue solid lines between atoms. The ligplots were amended by missing hydrogen bonds (gray broken lines) and hydrophobic contacts (red eyelashes) after manual inspection of the structural models of CytC-18 and CytC-78.

**Figure 7:**
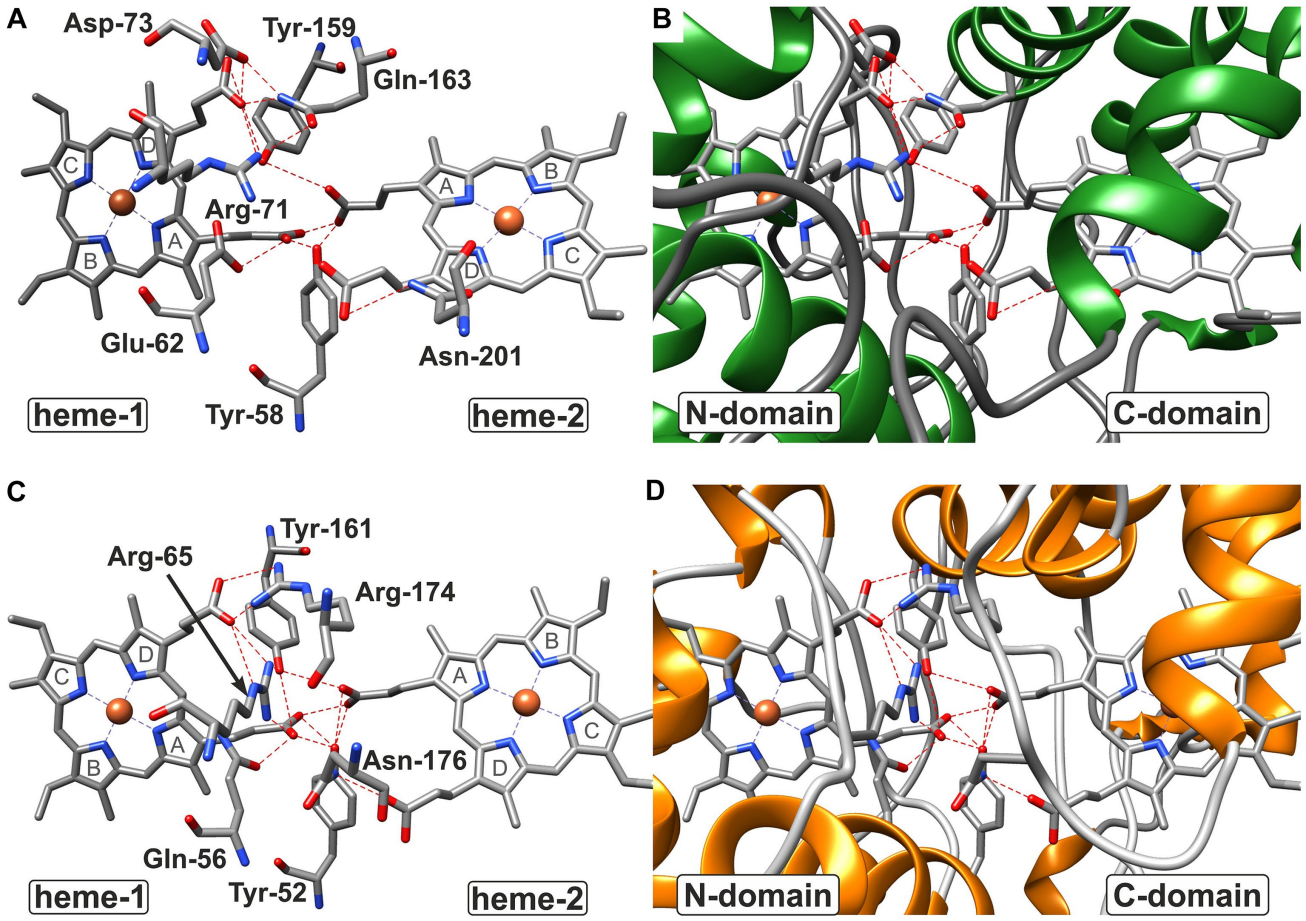
Hydrogen bond network at the domain:domain interface of CytC-18 **(A,B)** and CytC-78 **(C,D)**. The residues involved in hydrogen bonds at the domain:domain interface are indicated by three letter code and position number for CytC-18 **(A)** and CytC-78 **(C)** without the surrounding protein structure. The surrounding structure is shown for the same view at the interface colored by secondary structure. While coils are shown in gray, helices in are shown in green in CytC-18 **(B)** or in orange in CytC-78 **(D)**. Hydrogen bonds between residues and proprionate groups of the heme centers are indicated as red broken lines. The pyrrole ring numbering from **(A–D)** is indicated in gray letters for each heme center in **(A,C)**.

A typical feature of CytC-18, CytC-78, and other c_4_ cytochromes is the two-domain structure with heme-1 being bound within the N-terminal domain and heme-2 being bound in the C-terminal domain (Andersen et al., 2011; Ullrich et al., 2023). The heme-heme geometry in CytC-18 and CytC-78 is very similar to that of *P.s.* c_4_ and *A.f.* Cyc_41_ with the two heme centers being arranged in the same plane in a slightly tilted angle to each other (Ullrich et al., 2023). This geometry results in long Fe-Fe distances between the heme centers of 18.2 Å (CytC-18) and 19.1 Å (CytC-78) which is comparable to the distances in *A.f.* Cyc_41_ (18.7 Å) and *P.s.* c_4_ (19.2 Å), respectively, Table 3. We employed LigPlot^+^ to predict interdomain contacts using the dimplot feature (Figure 5) and protein:heme contacts in CytC-18 and CytC-78 (Figure 6). The dimplots in Figure 5 suggest that the interfaces of both cytochromes are stabilized by hydrophobic contacts and hydrogen bonds, whereas there are more hydrogen bonding residues in CytC-18 (11 vs. 8) and more hydrophobic contacts at the interface of CytC-78 (18 vs. 14). In both cytochromes these residues likely contribute to the arrangement of the proprionates of the pyrrole rings A within hydrogen bonding distance (Figure 7). The O-O distances between the O1A atoms of the contacting proprionate groups are 2 Å (CytC-18) and 2.4 Å (CytC-78), respectively, which is comparable to the O-O distances in *A.f.* Cyc_41_ (2.5 Å) and *P.s.* c_4_ (2.5 Å) (Table 3). Moreover, the analysis of the Ligplots (Figure 6) and the structural models (Figure 7) underline how the proprionate groups are involved in the hydrogen bond network in CytC-18 and CytC-78.

**Table 3:** Summary of structural properties of the domain:domain interfaces and the heme-binding pockets of *A. f.* Cyc_41_, CytC-18, CytC-78, and *P. s.* c_4_ based on LigPlot^+^ predictions and manual inspection of the structural models.

	*Acidithiobacillus ferrooxidans*	*Ferrovum* sp. PN-J47-F6		*Pseudomonas strutzeri*
	Cyc_41_	CytC-18	CytC-78	C_4_
*Heme geometry*				
domains	2	2	2	2
Fe-Fe distance	18.7 Å	18.2 Å	19.1 Å	19.2 Å
O-O-distance^1^	2.5 Å	2.0 Å	2.4 Å	2.5 Å
*Hydrogen bond network at the domain:domain-interface*				
Number per heme	Heme-1: 6 Heme-2: 4	Heme-1: 6 Heme-2: 3	Heme-1: 7 Heme-2: 3	Heme-1: 6 Heme-2: 6
Unbonded proprionate groups	Heme-2 D-ring (O1D)	Heme-2 D-ring (O2D)	Heme-2 D-ring (O1D)	none
Water molecules	7 (1 at heme-1 D-ring proprionate)	Not included in prediction	Not included in prediction	9 (2 at heme-2 D-ring proprionate)
*Nature of the binding pocket*				
Sum of hydrophobic contacts	Heme-1: 20 Heme-2: 19	Heme-1: 13 Heme-2: 12	Heme-1: 13 Heme-2: 12	Heme-1: 15 Heme-2: 12
Fraction of aromatic residues	Heme-1: 4 Heme-2: 3	Heme-1: 4 Heme-2: 3	Heme-1: 3 Heme-2: 3	Heme-1: 2 Heme-2: 2
*Heme surface accessibility*				
Heme-1	Partly accessible^2^	Partly accessible^2^	Accessible	Accessible
Heme-2	Accessible	Accessible	Accessible	Accessible

1 Distance between O1A-atoms of the heme proprionate groups.

2 Access only via nonpolar pyrrole rings.

In CytC-18, Arg-71 of the N-terminal domain forms hydrogen bonds to either Tyr-159 and Gln-163 of the C-terminal domain (Figure 5A). At the same time all three residues are in hydrogen bonding distance to the D-ring proprionate group of heme-1 (Figures 6A, 7A). Tyr-159 is also in hydrogen bonding distance to the proprionate group the A-ring of heme-2 (Figure 6B). Asp-73 forms a hydrogen bond to Gln-163 at the domain interface (Figure 5A) and to the A-ring proprionate of heme-1 (Figure 6A). Tyr-58 is involved in hydrophobic contacts at the domain interface (Figure 5A) and to heme-2 (Figure 6B) and forms hydrogen bonds to A-ring proprionates of heme-1 and heme-2 (Figure 6A). The hydrogen bond network in CytC-78 involves similar residues including Arg-65 of the N-terminal domain and Tyr-161 of the C-terminal domain forming hydrogen bonds at the domain interface (Figure 5B) and to the A-ring proprionates of heme-1 and heme-2 (Figures 6C,D, 7C). Tyr-52 forms a hydrogen bond to the A-ring proprionate of both hemes. Moreover, both tyrosines also provide hydrophobic contacts to heme-1 and heme-2, respectively (Figures 6C,D). Among the similar features in CytC-18 and CytC-78 are on the one hand the two tyrosine and one arginine residue pointing toward the proprionate contact site with their hydroxyl groups or guanidino group, respectively (Figure 7). On the other hand, in both cytochromes one of the oxygen atoms of the heme-2 D-ring proprionates remains without a hydrogen bonding partner (CytC-18: O2D, CytC-78: O1D). However, despite the general similarity of their the hydrogen bond networks, noteworthy unique features are two acidic residues (Glu-62, Asp-73) in CytC-18 in contrast to the basic Arg-174 in CytC-78.

The binding pockets of the heme centers were analyzed manually based on the prediction of hydrophobic contacts by LigPlot^+^ (Figure 6; Supplementary Figure 5). In CytC-18 both hemes show a more distinguished surrounding in their respective binding pockets (Supplementary Figures 5A–D) in comparison to the hemes in CytC-78 (Supplementary Figures 5E–H). Although the number of potential hydrophobic contacts is similar for both heme sites in CytC-18 (13 vs. 12), heme-1 appears to be more tightly surrounded by its contacts than heme-2. Of these hydrophobic contacts about a third is provided by aromatic residues in both binding-pockets (4 vs. 3). The heme centers in CytC-78 are surrounded by 13 and 12 hydrophobic contacts, respectively, of which 3 are aromatic in each of the binding pockets. In contrast to CytC-18, heme-2 appears to be more tightly surrounded by hydrophobic contacts provided by Met-150, Phe-151 and Ile-154 located in a loop region (Supplementary Figures 5G,H).

The high number of hydrophobic contacts in heme-binding sites contribute to the effective shielding of the heme centers from the access of surrounding solvent (Supplementary Figures 6A–D). Still, the D-ring proprionate groups of hemes-2 are accessible in both cytochromes. In CytC-18 this access site is surrounded by a large loop which might facilitate contact with an interaction partner during electron transfer (Supplementary Figure 6A). Heme-1 in CytC-18, however, seems to be accessible only via the nonpolar moiety of the C-ring. In CytC-78, on the other hand, both heme sites are accessible via the D-ring proprionates. Here, the heme-1 access site is dominated by basic residues (Arg-65 and Arg-174). At the heme-2 access site the hydrogen bond between Asn-176 (ND2) and the D-ring proprionate (O1D) is accessible.

#### 3.3.2 Comparison of the redox center environments of CytC-18 and CytC-78 to Cyc_41_ of *Acidithiobacillus ferrooxidans* and c_4_ of *Pseudomonas strutzeri*

In all four compared cytochromes the hydrogen bond network at the domain:domain interface involves arginine, tyrosine and glutamine residues forming hydrogen bonds to the proprionate groups (Figure 6; Supplementary Figure 7). For heme-1, one arginine, two tyrosines and one glutamine residue appear to be conserved while for heme-2 only two tyrosine residues are conserved in all four cytochromes. A common feature of the acidophile-derived cytochromes CytC-18, CytC-78 and *A.f.* Cyc_41_ is the exclusion of the one of the oxygen atoms of their D-ring proprionate at heme-2 from the hydrogen bond network (O1D in *A.f.* Cyc41 and CytC-78; O2D in CytC-18). Another common feature is the asymmetry of their hydrogen bond network with the heme-1 proprionates involving a higher number of contacts as well as a different set of residues in comparison to the heme-2 proprionates. In contrast to that the proprionate groups of the hemes in *P.s.* c_4_ similarly form hydrogen contacts to one arginine, one lysine, one glutamine and two tyrosines. Another unique feature of *P.s.* c_4_ are the lysine residues (Lys-42, Lys-148) (Supplementary Figures 7C,D) where the hydrogen bond networks of the acidophile-derived cytochromes include polar, but uncharged asparagine or glutamine residues or in case of CytC-18 even acidic glutamate and aspartate residues. Apart from *P.s.* c_4_, there are also the unique features in the other cytochromes such as the restriction of arginine residues to the heme-1 proprionates in CytC-18 and CytC-78, and the third tyrosine residue forming a hydrogen bond to heme-1 in *A.f.* Cyc_41_.

Comparison of the Ligplots of *A.f.* Cyc_41_ (Supplementary Figures 7A,B) and *P.s.* c_4_ (Supplementary Figures 7C,D), reveals the much higher number of hydrophobic contacts within the heme-binding pockets of *A.f.* Cyc_41_ (20 and 19) in comparison to *P.s*. c_4_ (15 and 13). The fraction of aromatic residues among these contacts is also higher in *A.f.* Cyc_41_ with 4 and 3, respectively, compared to 2 in each heme-binding pocket in *P.s*. c_4_. In CytC-18 and CytC-78 the number of hydrophobic contacts is comparable to those in *P.s*. c_4_, but the higher number of aromatic residues increases their fraction to one third of the total number of hydrophobic contacts in comparison to one fifth in *A.f.* Cyc_41_ and *P.s*. c_4_. These structural aspects might result in the extended shielding of the heme site from the surrounding solvent. While in the acidophile-derived cytochromes at least one of the two heme centers appears to be less accessible (heme-1 in *A.f.* Cyc_41_, heme-2 in CytC-78) or is even completely buried within the domain core (heme-1 in CytC-18), both heme centers in *P.s.* c_4_ are widely accessible (Supplementary Figures 6, 8). Also, the porphyrin moieties of the heme centers appear to be more tightly shielded in the three acidophile-derived cytochromes (Supplementary Figures 6, 8A,B) in comparison to *P.s*. c_4_ (Supplementary Figure 8C, D).

Despite the observed symmetry of the hydrogen bond network and the accessibility of the both heme sites in *P.s*. c_4_, the distribution of water molecules in hydrogen-bonding distance is different for the proprionates of the two heme centers (Supplementary Figures 8C, D). While seven water molecules are surrounding the proprionates of heme-2, there are only two in the case of heme-1 (Table 3). Thus, the distribution of water molecules within the hydrogen bond network at the domain:domain interface suggests that the actual solvent accessibility in *P.s.* c_4_ is also different for the two heme centers. This is similar for *A.f.* Cyc_41_ where the more buried heme-1 has only one water molecule in hydrogen-bonding distance in comparison to eight in case of the solvent accessible heme-2. Interestingly, these water molecules might form hydrogen bonds with the otherwise free O1D atom of heme-2 (Supplementary Figure 8B). Although, we cannot evaluate this observation for CytC-18 and CytC-78, it is possible that the different heme accessibility of the two domains might determine the respective electron transfer partner in the respiratory chain.

## 4 Discussion

Our combined approach of electrochemical characterization of the three *Ferrovum*-derived recombinant redox proteins HiPIP-41 and the c_4_ cytochromes CytC-18 and CytC-78 and their structural comparison to homologs of acidophiles and neutrophiles contributed to the field on two levels: First, the comparison of their redox behavior and their structural models revealed aspects of structure function correlations in CytC-18 and CytC-78 that present starting points for future mechanistic studies and that result in further hypotheses on their function the respiratory chain of *Ferrovum* sp. PN-J47-F6. Second, the *E_m_* of the three *Ferrovum*-derived redox proteins were found to be more positive than their neutrophile-derived homologs, and being at the same time more negative than the *E_m_*-values of homologs of more extreme acidophiles. Thus, our findings not only amend the results of earlier reports with electrochemical data on three further redox proteins, but moreover suggest a gradual fine-tuning of the *E_m_* in adaptation to the acidity of their host’s preferred habitats. Moreover, our structural comparisons revealed shared structural features of the acidophile-derived redox proteins that might be attributed with their more positive redox potentials.

### 4.1 Electrochemical characterization of CytC-18 and CytC-78 suggests anti-cooperative effects between their heme centers

The spectroelectrochemical characterization of CytC-18 and CytC-78 indicates that the full redox transition occurs stepwise via two redox transitions at different potentials. These lower and higher potential redox transitions are attributable to the two heme centers covalently bound in the N-terminal (heme-1) and the C-terminal domain (heme-2) of the cytochromes. In CytC-18, the Δ*E_m_* between the lower and higher potential heme is 140 mV while it is 91 mV in CytC-78. With exception of *P.a.* c_4_ (Carpenter et al., 2020) and Cyt c_4_ of *P. haloplanktis* (Di Rocco et al., 2008) the two heme centers in homologous cytochromes were also characterized by individual *E_m_*-values (Leitch et al., 1985; Cavazza et al., 1996; Giudici-Orticoni et al., 2000; Malarte et al., 2005; Chang et al., 2010; Carpenter et al., 2020). The two sequential redox transitions resulted in a double-sigmoidal shape of the absorption potential curves of CytC-18 and CytC-78 which was more clearly defined in case of CytC-18. Theoretically, this curve shape reflects the modeled redox behavior of a two-center redox protein with non-interacting *E_m_*-values (Catarino and Turner, 2001; Chi et al., 2010). However, for both cytochromes the lower potential redox transition only corresponded to approx. 25% of the total absorption change between the fully reduced and fully oxidized state while the higher potential redox transition corresponded to 75%. This observation suggests that the redox state of one heme center affects the *E_m_* of the other heme reflecting an anti-cooperative redox interaction (Zickermann et al., 1995) between the two heme centers in CytC-18 and CytC-78. Similar observations were also reported between hemes a and a_3_ in the cytochrome c oxidase of *Paracoccus denitrificans* (Gorbikova et al., 2006), hemes b and c_1_ in the *P. denitrificans* bc_1_ complex (Covian et al., 2007) or hemes b_558_ and b_595_ of the *E. coli* cytochrome bd oxidase (Bloch et al., 2009).

The analysis of the heme-heme geometry and the Fe-Fe distances highlighted a another common structural feature of CytC-18, CytC-78, *A. f.* Cyc_41_, and *P.s.* c_4_. Both heme centers are located in the same plane and in face-to-face orientation according the classification of de March et al. (2015). The spatial separation of the hemes in individual domains together with their face-to-face orientation leads to long distances Fe-Fe distances of 18.2 Å (CytC-18) and 19.2 Å (*P.s.* c_4_). This heme geometry affects the mode of electron transfer between the two redox centers. In the cytochrome c oxidase (Tan et al., 2004) or the decaheme outer membrane cytochromes MrtC and OmcA of *Shewanella oneidensis* (Tikhonova and Popov, 2014) the close proximity of the redox centers allows the rapid electron transfer via electron tunneling. Since this mode requires distances between the redox centers of less than 14 Å (Page et al., 1999), the electron transfer in CytC-18, CytC-78, *A. f.* Cyc_41_ and *P.s.* c_4_ is more likely to be realized via proton-coupled electron transfer (Huynh and Meyer, 2007). The slightly tilted angle of their hemes together with the face-to-face geometry results in the close proximity of the respective A-ring proprionates at the domain:domain interfaces. The four proprionate groups in each cytochrome are involved in a complex hydrogen bond network involving numerous residues of both domains. The experimentally solved structures of *P.s*. c_4_ and *A.f*. Cyc_41_ also included water molecules at the domain:domain interface which are also likely to be part of this hydrogen bond network. These hydrogen bond networks in all four c_4_ cytochromes present the pre-requisite of the proton-coupled electron transfer in a pre-association phase by minimizing the proton tunneling distance (Huynh and Meyer, 2007).

### 4.2 The character of the interdomain contacts in CytC-18 and CytC-78 might contribute to their different redox behavior

The constitution of the hydrogen bond network at the domain:domain interface might affect the redox kinetics of the cytochromes (Crowley and Ubbink, 2003). Although we did not determine kinetics parameters in the present study our electrochemical characterization of the *Ferrovum*-derived cytochromes strongly suggests that CytC-18 and CytC-78 harbor individual redox behaviors as was indicated by the different potential windows necessary for the full redox transition and the different degree of hysteresis between the oxidative and the reductive potential sweeps. The overall constitution of hydrogen bond network is very similar in CytC-18 and CytC-78. However, the most prominent differences present the acidic aspartate and glutamate residues in proximity of the heme-1 proprionate groups in CytC-18. They are expected to be fully protonated at pH 3.1 and are thus uncharged but also potential donors and acceptors of hydrogen bonds. In contrast to that, the hydrogen bond network of the heme-1 proprionate groups in CytC-78 involves an additional arginine residue which is not only a potential hydrogen bonding partner but also carries a positive charge at the guanidino group. Apart from the influence of the hydrogen bond network on the interdomain electron transfer also the dynamics of the domain:domain association is thought to play a central role for the redox activity of a protein (Camacho et al., 1999; Crowley and Ubbink, 2003). Indeed we observed differences in the nature of contacts at the domain:domain interfaces in CytC-18 and CytC-78. While the domain interface of CytC-18 involves more hydrogen bonds than the interface in CytC-78, the situation is vice versa with respect to the number of hydrophobic contacts. Since hydrophobic contacts act in longer range than hydrogen bonds (Israelachvili and Pashley, 1982; Onofrio et al., 2014) the different nature of interdomain contacts in the two *Ferrovum*-derived cytochrome is probably affecting the association dynamics and thereby the intramolecular electron transfer kinetics.

### 4.3 The constitution of the cofactor binding pockets appears to be the major influencing factor for the more positive *E_m_* in acidophile-derived C_4_ cytochromes

In their comparative study on structure and function correlations involving 33 heme-binding proteins with experimentally solved structures Smith et al. (2010) did not identify clear connections between the cytochrome’s *E_m_* and specific structural features. They have instead postulated that a combination of various structural features influence the E_m_. We focused our structural comparison therefore on hydrophobic contacts in the heme binding pockets and heme solvent accessibility, because they have already been identified as a central *E_m_* influencing factor in other redox proteins (Di Rocco et al., 2008, 2011; de March et al., 2015; Hosseinzadeh et al., 2016; Carpenter et al., 2020). The structural comparison of CytC-18, CytC-78, *A.f.* Cyc_41_, and *P.s*. c_4_ revealed that the heme centers in *A.f.* Cyc_41_ formed the highest number of hydrophobic contacts to their binding pockets. The total number of hydrophobic contacts in the other three cytochromes was similar, but CytC-18 and CytC-78 harbored the largest fraction of aromatic residues among these contacts. During the purification and concentration procedure of CytC-78 and CytC-18 we indeed observed a long stability of the fully reduced state of CytC-78 or partly reduced state of CytC-18 (Ullrich et al., 2023), respectively, while *P.s.* c_4_ was observed to quickly oxidize during the crystallization process in absence of an chemical oxidant (Kadziola and Larsen, 1997). Thus, the higher number of hydrophobic contacts or aromatic residues in proximity of the heme center might indeed result in the more effective shielding of the heme centers in the acidophile-derived cytochromes as was suggested by their structural models. However, the domain:domain interface, and thereby the hydrogen bond network involving the heme proprionates and residues of both domains, appears to be equally accessible for solvent water molecules since the number of water molecules at the interface of *A.t.* Cyc_41_ (7) and *P.s.* c_4_ (9) was similar.

Besides the above discussed contacts in the heme-binding pocket, other structural aspects were discussed as influencing factors of the *E_m_*, such as non-planar distortions of the heme porphyrin ring that have also been identified to effect the heme’s redox properties by influencing the *E_m_*, transition dipoles and axial ligand affinity (Shelnutt et al., 1998; Smith et al., 2010). In case of *A.f.* Cyc_41_ and *P.s.* c_4_ no distortion of the porphyrin ring structure has been reported (Kadziola and Larsen, 1997; Abergel et al., 2003; Carpenter et al., 2020). An overall structural similarity of CytC-18 and CytC-78 to *A.f.* Cyc_41_ and *P.s.* c_4_ leads us assume that the heme centers in the *Ferrovum*-derived cytochromes do also not have any structural distortions. Furthermore, mutant studies demonstrated that the presence of polar or charged residues in close proximity to the heme iron decreased the E_m_. The disruption of a hydrogen bond to the histidine axial ligand in myoglobin increased its *E_m_* (Bhagi-Damodaran et al., 2014). This effect was explained by the increase of the positive character of the Nε-atom of the histidine axial ligand and the thereby reduced electron donating ability of its imidazole ring toward the heme iron (Valentine et al., 1979). The exchange of aliphatic residues providing hydrophobic contacts to the porphyrin ring system by acidic or polar residues similarly resulted in the *E_m_* becoming up to 200 mV more negative than in the wildtype myoglobin (Varadarajan et al., 1989). Specific electrostatic contacts between the protein chain and the heme center indeed present interesting starting points for future studies. These will also require experimental structure determination because electrostatic interactions require a close proximity of the partners, and thus a high-resolution structure of the protein is needed to study them.

### 4.4 The more positive *E_m_*-values in acidophile-derived HiPIPs might be attributed to additional cysteine residues

The *E_m_* of HiPIP-41 is approx. 240 mV more positive than that of HiPIP 1Isu of *R. tenuis* and similar to the *E_m_* of Hip of *A. ferridurans*. The covalent binding of the [4Fe-4S]-cofactor by four highly conserved cysteines resulting in its tight shielding from the solvent is acknowledged to cause the far more positive *E_m_* in comparison to ferredoxins (Carter et al., 1972; Dey et al., 2007). Moreover, fewer hydrogen bonds between peptide bonds of the protein backbone and the cysteine sulfur atoms were detected in HiPIPs in contrast to ferredoxins (Backes et al., 1991), resulting in a lower polarity of the cofactor environment in HiPIPs (Heering et al., 1995). Beside the similar electrostatic contacts in the cofactor environment, the total number of hydrophobic contacts in the cofactor binding pocket was also similar in the structural models of HiPIP-41, HiPIP 1Isu and Hip, resulting in the anticipated complete burial of their cofactors and the effective shielding from the surrounding solvent. Moreover, the superimposition of the three structural models showed a high structural similarity and the conserved positions of the hydrophobic contacts. Since aromatic residues are anticipated to contribute to the cofactor stability by supporting its tight shielding from the protein’s surrounding solvent (Agarwal et al., 1995; Iwagami et al., 1995), their high level of conservation was not surprising. However, with respect to non-aromatic residues only their position within the protein sequence and structure were conserved but not the residues themselves. In this context, we did not identify any correlations between the size or the degree of hydrophobicity of the hydrophobic contacts and the E_m_.

However, the most remarkable structural feature of HiPIP-41 and Hip present the two additional cysteine residues. In Hip, Cys-52 and Cys-84 form a disulfide bridge which was assumed to contribute to the acid stability of the protein (Nouailler et al., 2006). In HiPIP-41, Cys-50 and Cys-55 are located in the core of the protein and Cys-53 is oriented toward the cofactor. These two additional cysteines were found to be highly conserved in the HiPIPs of *Ferrovum* spp., but they are not found in homologous proteins in neutrophilic relatives (Ullrich et al., 2023). Since cysteine residues were classified as very hydrophobic due to their very frequent location within a protein’s hydrophobic core (Janin, 1979; Rose et al., 1985), we propose that they might indeed contribute to the hydrophobic character of the surrounding of the [4Fe-4S]-cofactor and thereby modulating the *E_m_* in the acidophile-derived HiPIPs. The HiPIP Iro from *A. ferrooxidans* with a very positive *E_m_* of 630 mV (Yamanaka and Fukumori, 1995), however, does also not contain any additional cysteine residues (Nouailler et al., 2006).

Apart from the solvent accessibility of the [4Fe-4S]-cofactor and the very hydrophobic character of its binding pocket, also the polarity of the cluster environment (Heering et al., 1995; Stephens et al., 1996), the general surface charge of the protein (Banci et al., 1995; Stephens et al., 1996; Capozzi et al., 1998) and the position of polar patches on the protein’s surface (Babini et al., 1998; Parisini et al., 1999) have been discussed as *E_m_* modulating factors. The surface of HiPIP 1Isu is characterized by the presence of patches with both negative and positive electrostatic potential caused by acidic, basic and polar residues being exposed to the surface (Rayment et al., 1992). In contrast, the surface of HiPIP-41 is characterized by large patches of neutral surface electrostatic potential or patches with slightly positive electrostatic potentials (Ullrich et al., 2023). With respect to missing data on the surface charge of Hip it is difficult to evaluate its potential influencing effect of the overall surface charge on the E_m_. An earlier study of Babini et al. (1998) has indeed demonstrated how the mutation of a surface exposed histidine influences the *E_m_* and the redox kinetics of the HiPIP of *Chromatium vinosum*. Residues Tyr-54 and Asn-27 in HiPIP-41 might present candidates for similar studies since they are in contact with both, the cofactor-binding pocket and the surface of the protein.

### 4.5 Implications on the organization of the electron transfer chain in *Ferrovum* sp. PN-J47-F6

Based on our structural analyses and comparisons, we propose that the N-terminal hemes (hemes-1) in CytC-18 and CytC-78 are the higher potential hemes and the C-terminal hemes (hemes-2) the lower potential hemes. In CytC-18, heme-1 is more tightly surrounded by aliphatic and aromatic residues resulting in its deep burial within the protein core and an efficient shielding from the surrounding solvent. Similar observations were reported for the N-terminal heme center of Cyc_41_ (Abergel et al., 2003) and the C-terminal heme center of *P.s.* c_4_ (Nissum et al., 1997; Carpenter et al., 2020), respectively. Moreover, we assume that the tighter shielding of the N-terminal heme center in CytC-18 in comparison to the C-terminal heme results in the observed larger Δ*E_m_* by increasing the *E_m_* of the higher potential heme in comparison to the lower potential heme. The heme binding-pockets in the N- and C-terminal domains in CytC-78 have a more similar appearance which we assume contributes to the smaller Δ*E_m_* of its hemes. Still, the overall similar number and nature of hydrophobic contacts in the heme-binding pockets of both *Ferrovum*-derived cytochromes lead us to infer that the higher potential heme is associated with the N-terminal domain in both cytochromes.

The different location of the higher and lower potential hemes in the acidophile-derived c_4_ cytochromes CytC-18, CytC-78, and *A. f.* Cyc_41_ in contrast to *P.s.* c_4_ might reflect the different organization of the electron transfer chains. While CytC-18, CytC-78, and Cyc_41_ accept electrons from a soluble redox protein with a very positive *E_m_* (HiPIP-41 or rusticyanin) to transfer them further downhill to the cytochrome c oxidase in the inner membrane (Giudici-Orticoni et al., 1999; Ullrich et al., 2023), *P.s.* c_4_ shuttles electrons between inner membrane complexes III and IV (Smith et al., 1981; Kadziola and Larsen, 1997). The C-terminal domain of *P.s.* c_4_ was proposed to interact with the cytochrome c oxidase (Iwata et al., 1995; Tsukihara et al., 1995) suggesting that the higher potential heme transfers electrons further downhill to the terminal oxidase. A mutant study of Cyc_41_ variants (Malarte et al., 2005) and a computational docking study (Jiang et al., 2021) draw a similar conclusion for the interaction of Cyc_41_ in the electron transfer chain in *A. ferrooxidans*, suggesting that the C-terminal lower potential heme accepts electrons from rusticyanin while the N-terminal higher potential heme transfers them downhill to the aa_3_-type cytochrome c oxidase. Expanding this conclusion to CytC-18 and CytC-78, we propose that the C-terminal domains with the lower potential hemes interact with HiPIP-41 in the electron transfer chain of *Ferrovum* sp. PN-J47-F6.

With respect to the electron acceptors of CytC-18 and CytC-78 only educated guesses are possible at this stage of our current research. In *A. ferrooxidans*, Cyc_41_ (Cyc_1_) has a slightly more positive *E_m_* than CycA1 (Cavazza et al., 1996; Giudici-Orticoni et al., 2000). While Cyc_41_ transfers electrons downhill to the aa_3_-type cytochrome c oxidase, CycA1 transfers a smaller fraction of electrons uphill to the bc_1_ complex (Bird et al., 2011). The encoding gene of Cyc_41_ is localized directly downstream of the gene encoding the outer membrane monoheme cytochrome Cyc2 (Valdés et al., 2008). In *Ferrovum* sp. PN-J47-F6, the gene coding CytC-18 or its homolog in *Ferrovum* spp. is localized directly downstream of the Cyc2-like encoding gene (Ullrich et al., 2018). Based on the genetic organization of its encoding gene and its slightly more positive *E_m_*, we propose that CytC-18 transfers electrons downhill to the ccb_3_-type cytochrome c oxidase while CytC-78 interacts with the bc_1_ complex within the uphill branch of the electron transfer chain.

## Data availability statement

The raw data supporting the conclusions of this article will be made available by the authors, without undue reservation.

## Author contributions

SRU: Conceptualization, Data curation, Formal analysis, Funding acquisition, Investigation, Methodology, Project administration, Resources, Software, Supervision, Validation, Visualization, Writing – original draft, Writing – review & editing. HF: Writing – original draft, Writing – review & editing. CA-G: Writing – original draft, Writing – review & editing.
