# Supplementary figures and images for: Electrochemical and structural characterization of recombinant respiratory proteins of the acidophilic iron oxidizer Ferrovum sp. PN-J47-F6 suggests adaptations to the acidic pH at protein level
Source: Front Microbiol. 2024 Feb 7;15:1357152. doi: 10.3389/fmicb.2024.1357152 (PMC10879576; doi:10.3389/fmicb.2024.1357152)

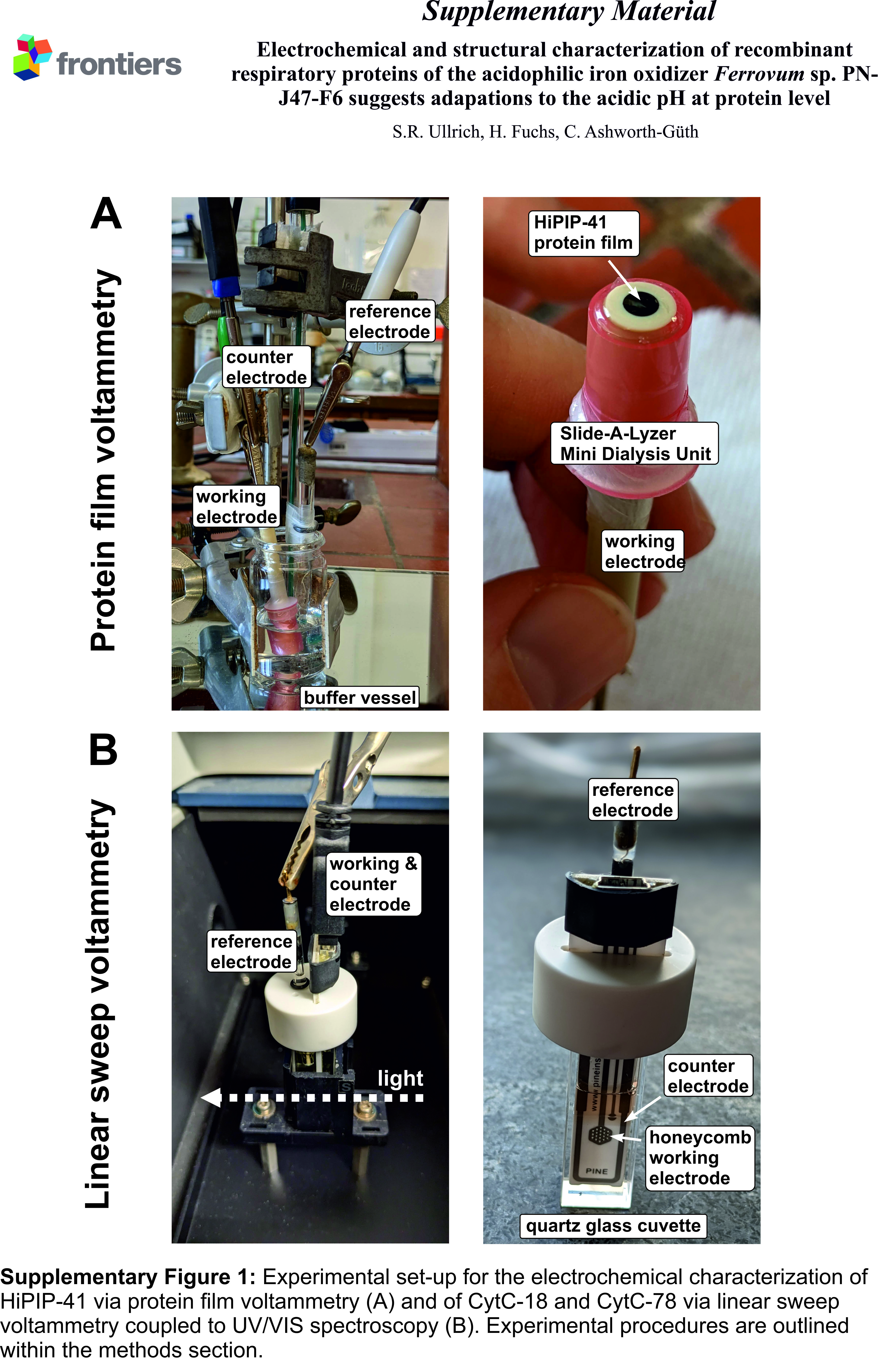

Supplement: Supplementary file 1 [file Image_1.jpg]

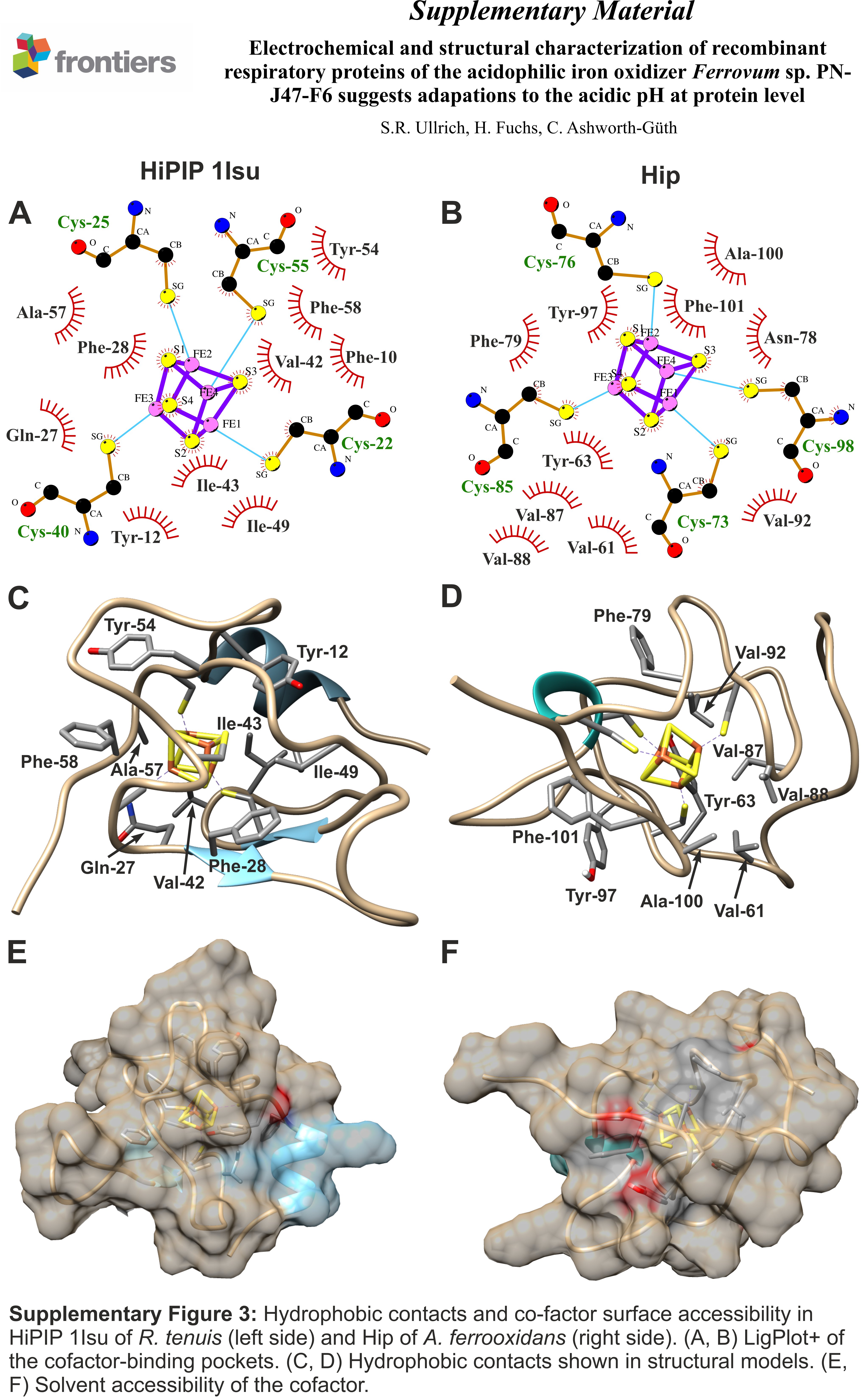

Supplement: Supplementary file 3 [file Image_3.JPEG]

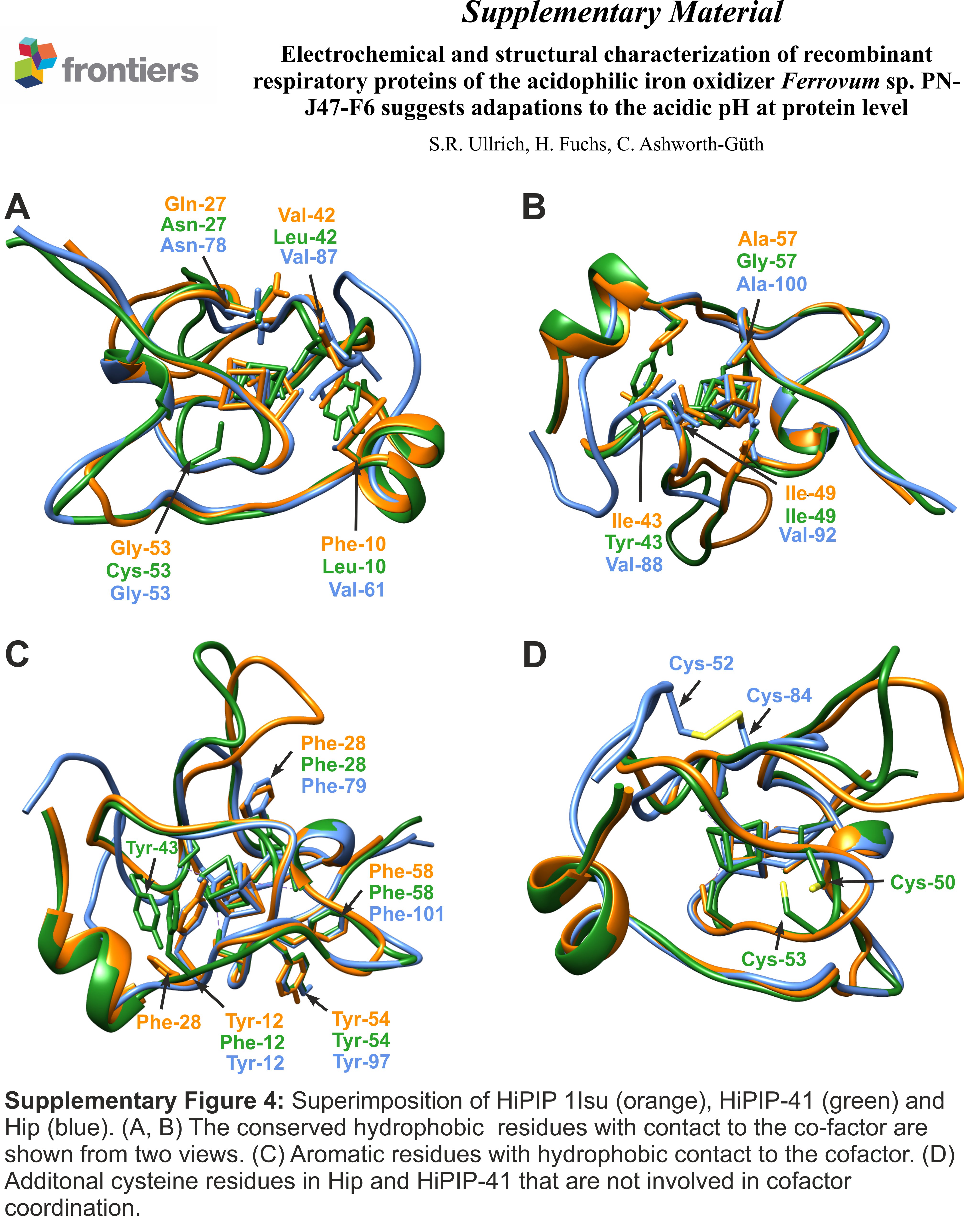

Supplement: Supplementary file 4 [file Image_4.JPEG]

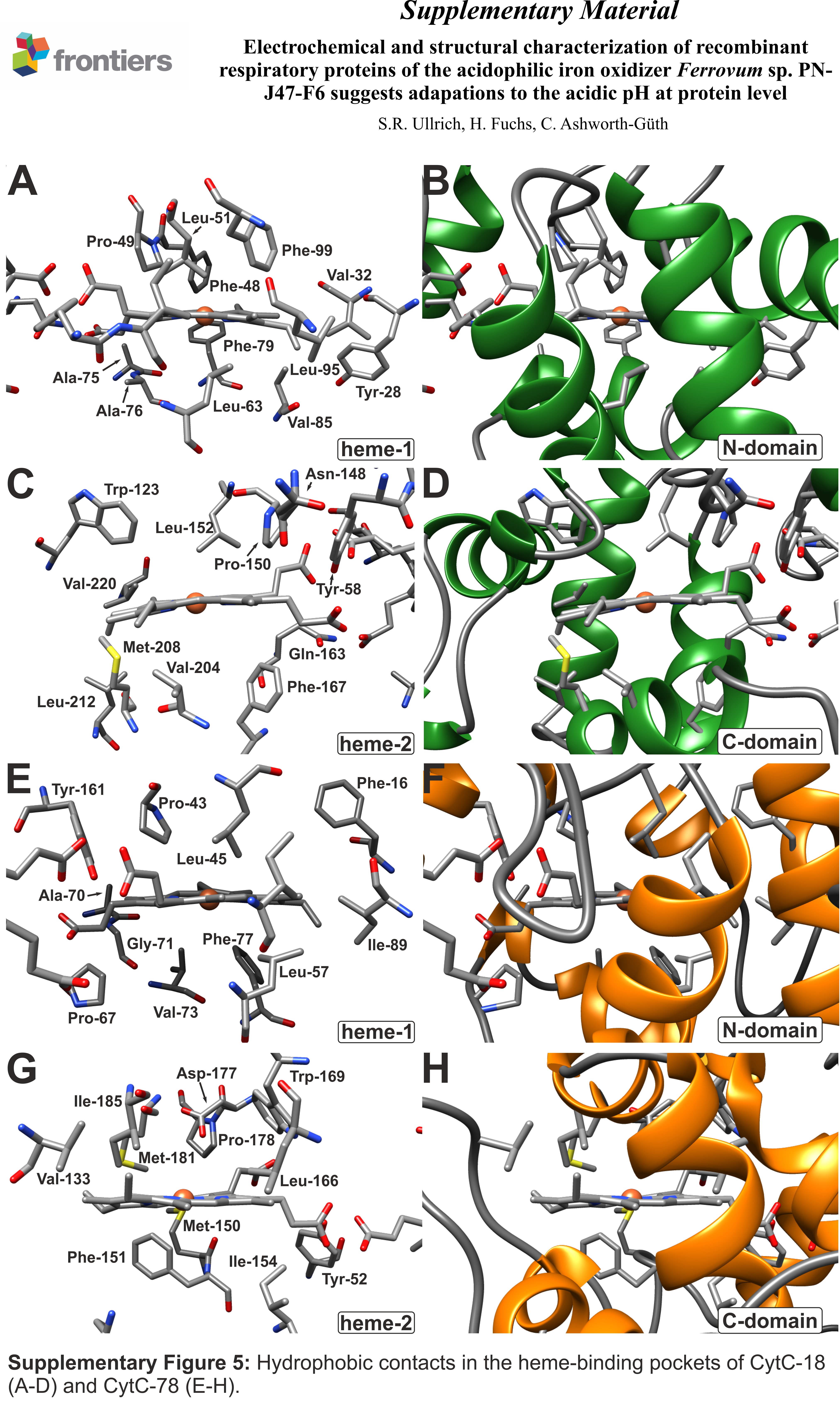

Supplement: Supplementary file 5 [file Image_5.JPEG]

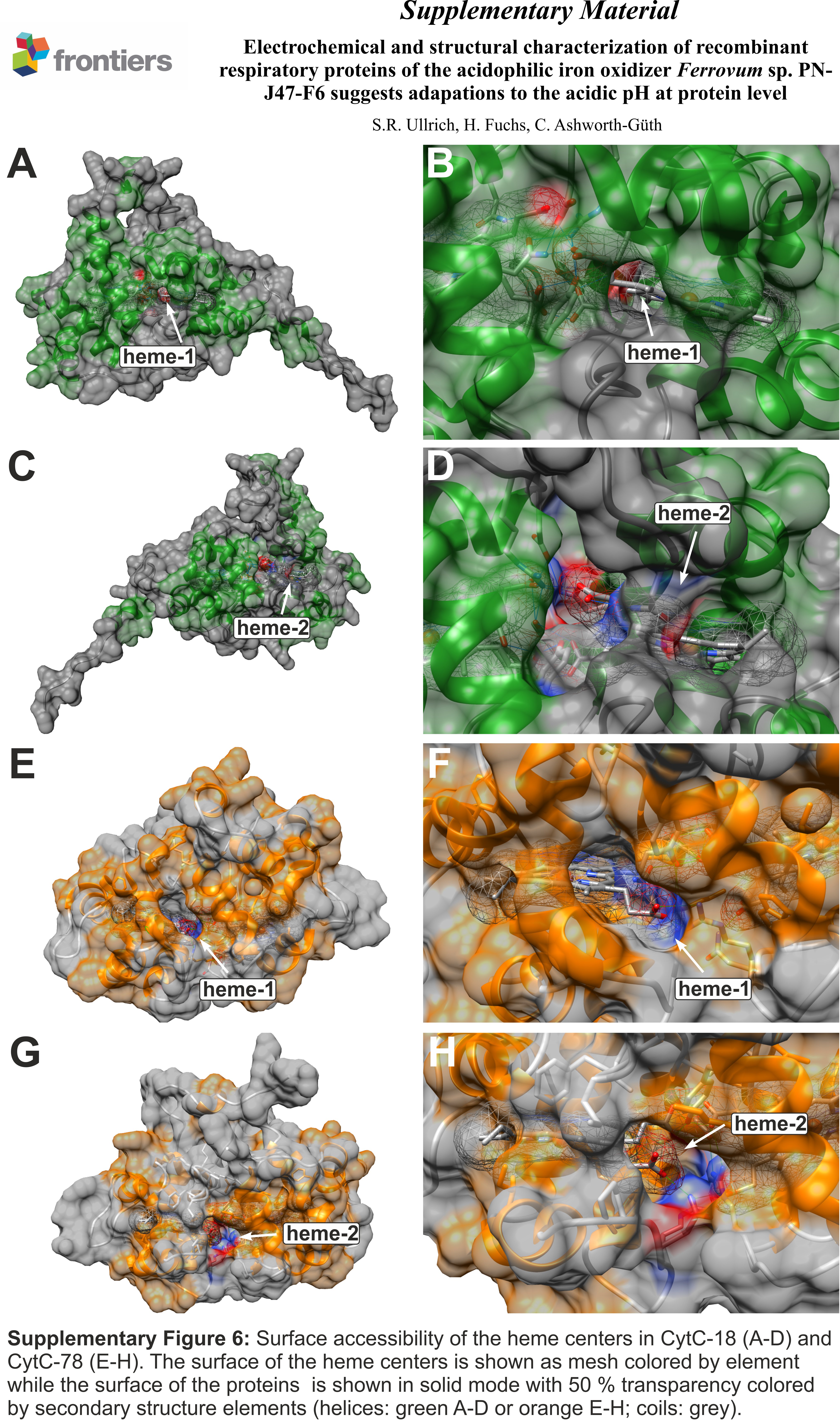

Supplement: Supplementary file 6 [file Image_6.JPEG]

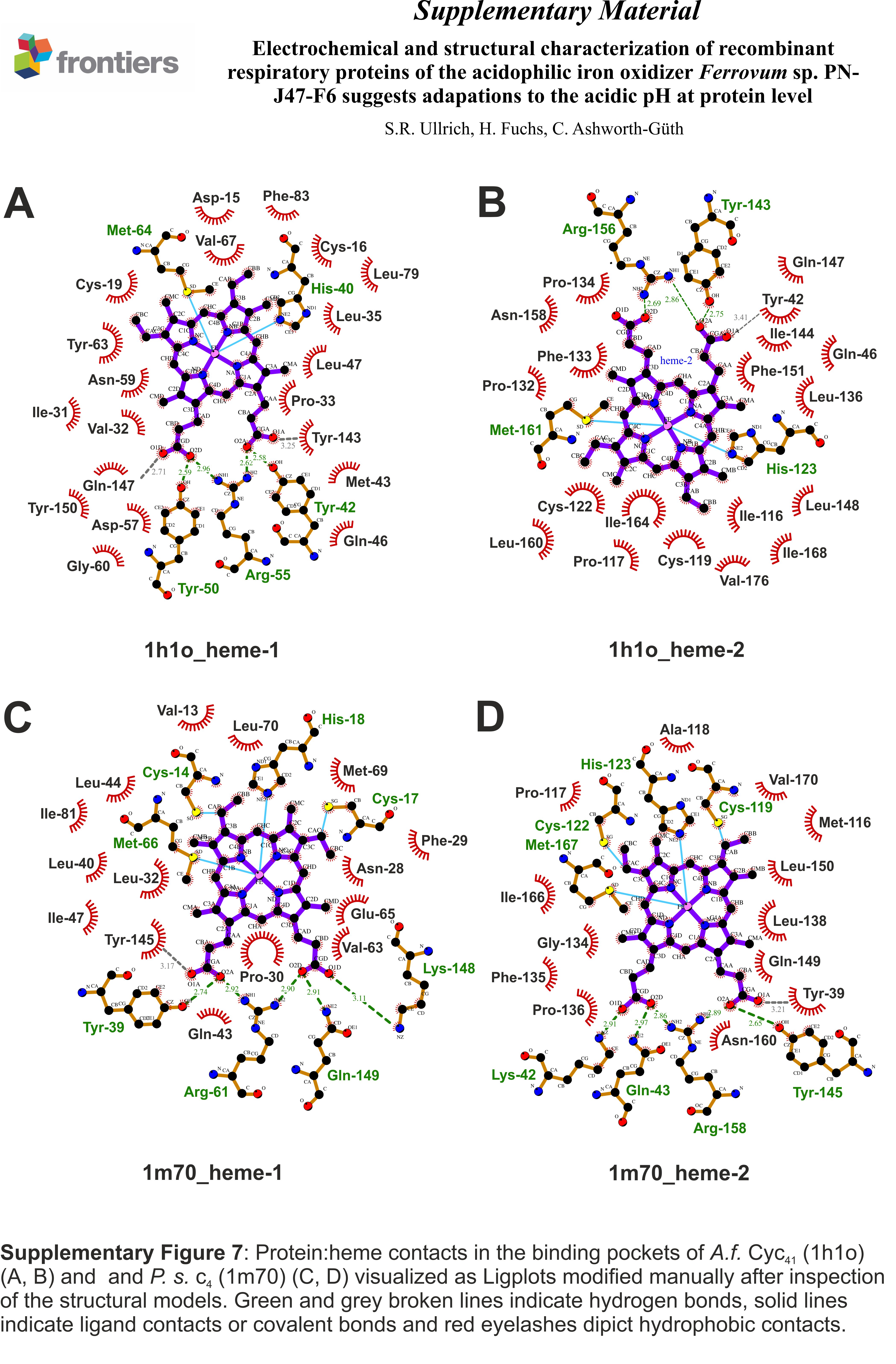

Supplement: Supplementary file 7 [file Image_7.jpg]

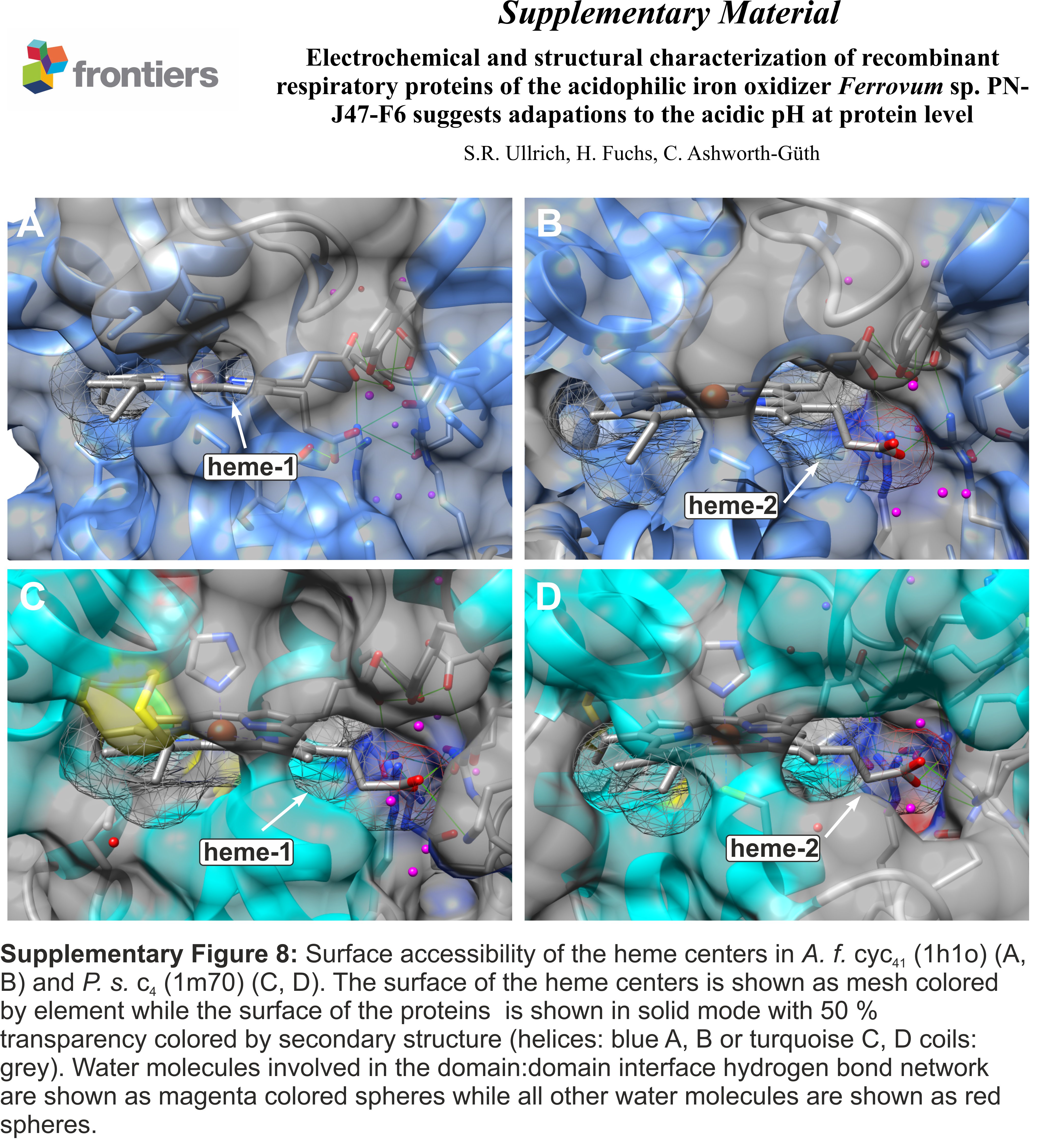

Supplement: Supplementary file 8 [file Image_8.jpeg]
