# Supplementary material for: Electrochemical and structural characterization of recombinant respiratory proteins of the acidophilic iron oxidizer Ferrovum sp. PN-J47-F6 suggests adaptations to the acidic pH at protein level
Source: Front Microbiol. 2024 Feb 7;15:1357152. doi: 10.3389/fmicb.2024.1357152 (PMC10879576; doi:10.3389/fmicb.2024.1357152)
Supplement: Supplementary file 2 [file Image_2.pdf]

Supplementary Material

Electrochemical and structural characterization of recombinant respiratory proteins of the acidophilic iron oxidizer *Ferrovum* sp. PN-J47-F6 suggests adapations to the acidic pH at protein level

S.R. Ullrich, H. Fuchs, C. Ashworth-Güth

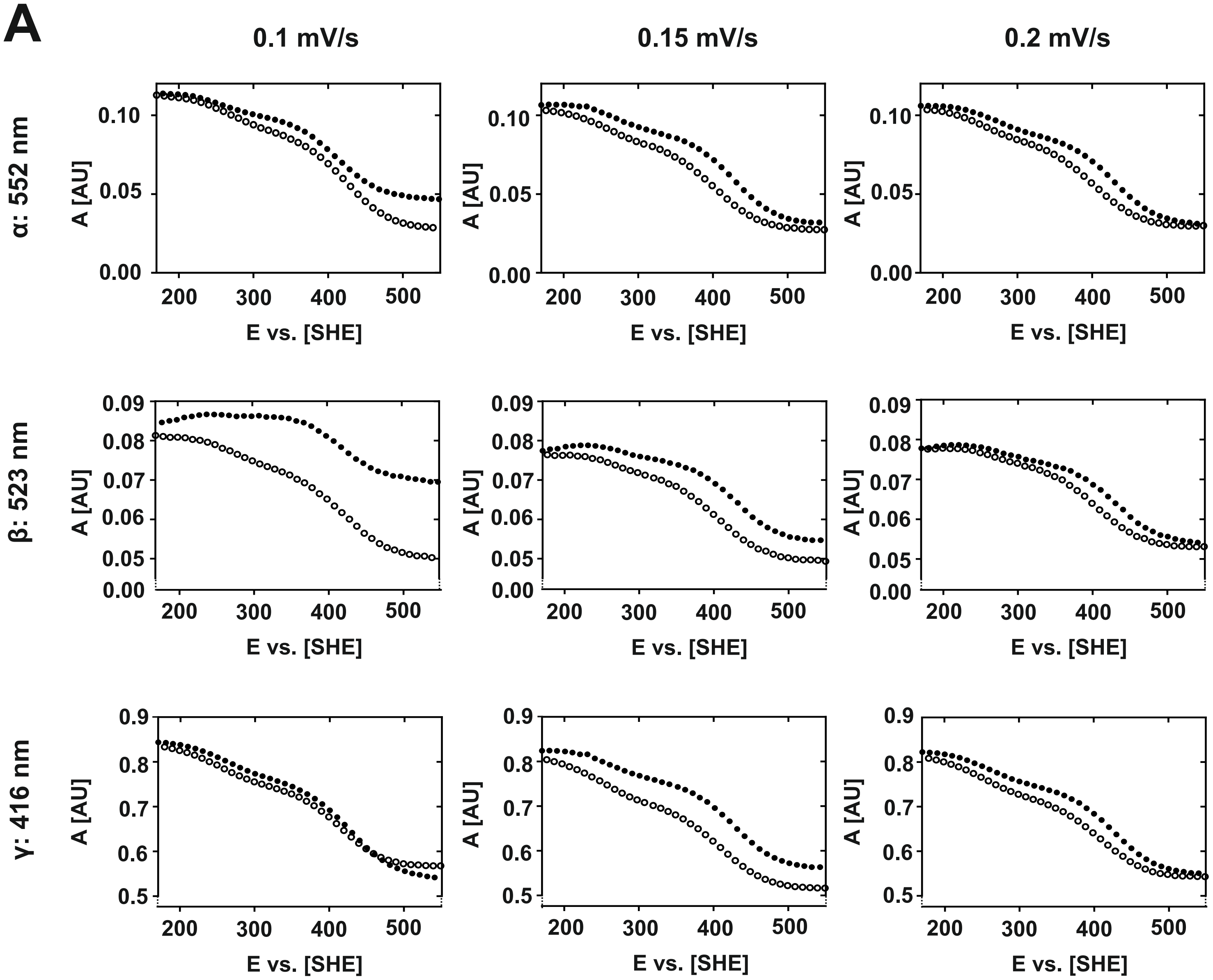

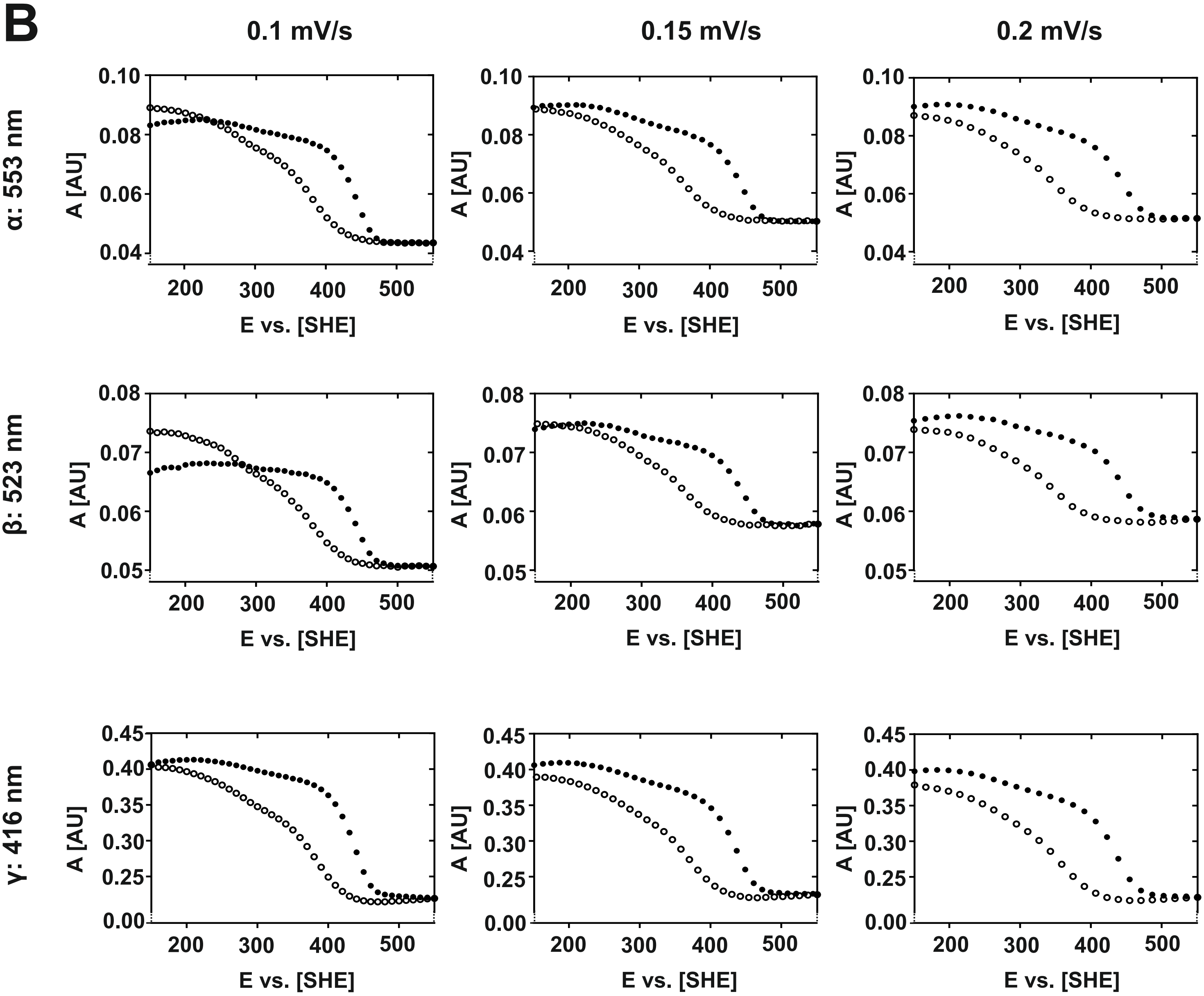

Supplementary Figure 2: Absorption intensities of the α-, β- and γ-peaks of CytC-18 (A) and CytC-78 (B) plotted against the applied potentials during oxidative (closed circles) and reductive sweeps (open circles). The potential sweep rates were varied between 0.1, 0.15 and 0.2 mV/s within the potential limits of 190 and 550 mV vs. SHE. For CytC-18, absorption intensities were recorded every 10 mV. For CytC-78, absorption intensities were recorded every 10 mV (0.1 mV/s), 12 mV (0.15 mV/s) or 16 mV (0.1 mV/s), respectively.
